# Analysis of a multi-type resurgence of *Mycobacterium bovis* in cattle and badgers in Southwest France, 2007-2019

**DOI:** 10.1186/s13567-023-01168-8

**Published:** 2023-05-03

**Authors:** Malika Bouchez-Zacria, Sandrine Ruette, Céline Richomme, Sandrine Lesellier, Ariane Payne, Maria-Laura Boschiroli, Aurélie Courcoul, Benoit Durand

**Affiliations:** 1 Laboratory for Animal Health, French Agency for Food, Environmental and Occupational Health and Safety (ANSES), University Paris-Est, 14 Rue Pierre Et Marie Curie, 94700 Maisons-Alfort, France; 2 French Office for Biodiversity (OFB), Research and Scientific Support Direction, Vincennes, France; 3 Nancy Laboratory for Rabies and Wildlife, French Agency for Food, Environmental and Occupational Health and Safety (ANSES), Malzéville, France; 4 Tuberculosis Reference Laboratory, Bacterial Zoonosis Unit, Laboratory for Animal Health, Paris-Est University, ANSES, 94700 Maisons‑Alfort, France; 5 Oniris, Nantes, France; 6 Independent Researcher, Audincthun, France

**Keywords:** *Mycobacterium bovis*, cattle, *Meles meles*, epidemic model, multi-host, multi-type, reproduction number, generation time

## Abstract

Although control measures to tackle bovine tuberculosis (bTB) in cattle have been successful in many parts of Europe, this disease has not been eradicated in areas where *Mycobacterium bovis* circulates in multi-host systems. Here we analyzed the resurgence of 11 M*. bovis* genotypes (defined based on spoligotyping and MIRU-VNTR) detected in 141 farms between 2007 and 2019, in an area of Southwestern France where wildlife infection was also detected from 2012 in 65 badgers. We used a spatially-explicit model to reconstruct the simultaneous diffusion of the 11 genotypes in cattle farms and badger populations. Effective reproduction number *R* was estimated to be 1.34 in 2007–2011 indicating a self-sustained *M. bovis* transmission by a maintenance community although within-species *R*s were both < 1, indicating that neither cattle nor badger populations acted as separate reservoir hosts. From 2012, control measures were implemented, and we observed a decrease of *R* below 1. Spatial contrasts of the basic reproduction ratio suggested that local field conditions may favor (or penalize) local spread of bTB upon introduction into a new farm. Calculation of generation time distributions showed that the spread of *M. bovis* has been more rapid from cattle farms (0.5–0.7 year) than from badger groups (1.3–2.4 years). Although eradication of bTB appears possible in the study area (since *R* < 1), the model suggests it is a long-term prospect, because of the prolonged persistence of infection in badger groups (2.9–5.7 years). Supplementary tools and efforts to better control bTB infection in badgers (including vaccination for instance) appear necessary.

**Supplementary Information:**

The online version contains supplementary material available at 10.1186/s13567-023-01168-8.

## Introduction

*Mycobacterium bovis*, belonging to the *Mycobacterium tuberculosis* complex, is the main causal agent of bovine tuberculosis (bTB). The geographic distribution of the five *M. bovis* clonal complexes [1, 2] (i.e. “groups of strains all descended from a single cell that was the most recent common ancestor (MRCA) of the clonal complex and all bearing characteristics derived from the MRCA” [3]) is related to the trade of cattle as the main host of bTB, in comparison with other susceptible livestock species such as sheep [4], goats [5], and swine [6]. In several European countries, surveillance and control programs of bTB in farm animals started in the 1950’s. Initially focused on cattle farms, they have been successful at eradicating bTB in many parts of Europe. However, in more complex epidemiological systems involving several species, bTB is still prevalent. In some areas, these multi-host epidemiological systems include cattle farms and other domestic [7] or/and wild mammal species, such as European badger (*Meles Meles*), wild boar (*Sus scrofa*), red deer (*Cervus elaphus*), fallow deer (*Dama dama*), roe deer (*Capreolus capreolus*), or red fox (*Vulpes vulpes*) [8–11].

**Figure 1:**
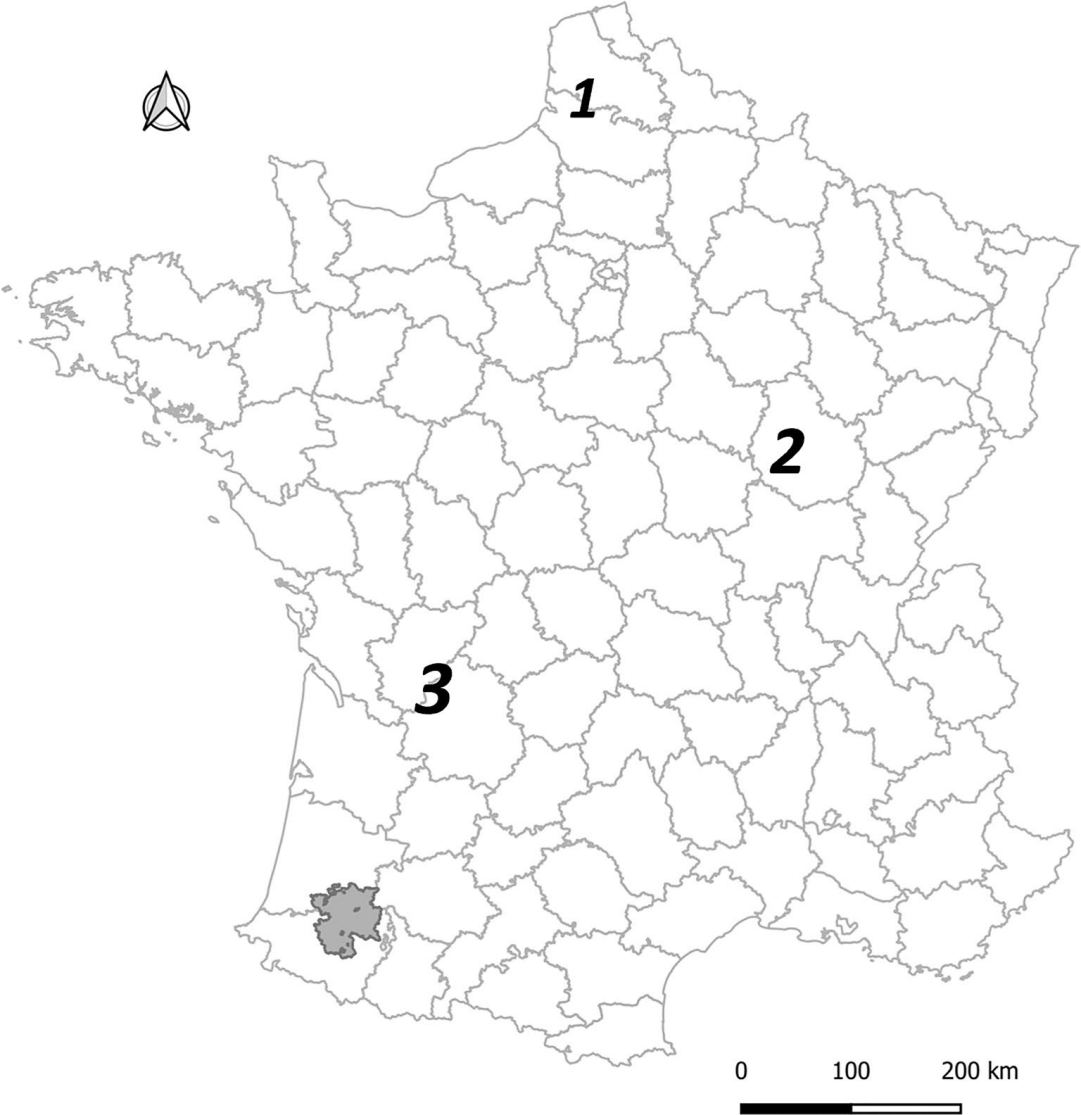
**Location of the main areas where *M. bovis*** **has circulated or is circulating in cattle and wildlife in France.** Grey area: location of the study area in Pyrénées-Atlantiques – Landes; 1: Brotonne forest; 2: Côte d’Or; 3: Dordogne-Charente; see [16] for more details.

The extent of *M. bovis* circulation in wildlife and the contribution of different species to this circulation may vary between infected areas. In Europe, badgers have been shown to be maintenance hosts in parts of the United Kingdom and Ireland [12], whereas in the south of the Iberian Peninsula, wild boars are mostly considered responsible for the persistence of *M. bovis* [13]. However, given the slow spread of *M. bovis* within and between infected populations, and the cryptic nature of bTB limiting diagnostic sensitivity, one should remain cautious while interpreting local epidemiological data and categorizing wild spillovers from actual reservoirs. In France, the circulation of *M. bovis* in wildlife was identified for the first time in 2001 in the Brotonne forest, Normandy, mostly in red deer and wild boar, and rarely in badgers [14, 15]. Cases in badgers were also detected in 2009 in Côte d’Or and in 2010 in Dordogne-Charente [14, 16] (Figure 1). Following the implementation in 2011 of a national surveillance system for *M. bovis* in free-ranging wildlife (“Sylvatub”), 11 “high-risk areas” (including the Brotonne forest), with *M. bovis* infection in wildlife, were identified. In contrast with the Brotonne forest where the high bTB prevalence and severe lesions in red deer suggested a self-sustaining epidemic in this species [15, 17], the ten new “high-risk areas” presented bTB infected wildlife mostly in badgers and wild boar, and to a lesser extent red deer, roe deer, and/or red foxes in 2019 [10, 11, 16]. In these populations, the self-sustained nature of *M. bovis* transmission and the epidemiological roles of different wild hosts remain unclear, and are probably variable according to local epidemiological systems and conditions.

The reproduction number *R* (also called net or effective reproduction number) is the average number of successful transmissions per epidemiological unit [18]. Usually computed using individuals as epidemiological units, *R* can also be calculated at the population level. In this case, a multi-host system is composed of animal populations of several species (e.g. cattle herds and badger social groups), between which two types of transmission routes can be distinguished: intra-species, i.e. between populations of the same species, and inter-species, i.e. between populations of different species. Estimating *R* separately for these different transmission routes helps disentangling the respective importance of different species in the overall transmission dynamics. When the intra-species *R* is > 1, populations of this species are considered as a reservoir, therefore able to maintain the pathogen without external source and to transmit the pathogen to another population (target). In another situation, when the overall *R* is > 1 but all the intra-species *R*s are < 1, the entire multi-host system acts as a maintenance community, but not the specific populations [19–24]. The generation time is the delay between successive infections in a chain of transmissions between epidemiological units [18]. Like *R*, it can be calculated using individuals or populations as epidemiological units. In the latter case, in a multi-hosts system, the estimation of the generation time for each transmission route indicates whether transmission occurs more rapidly between populations of the same species than between populations of different species.

Spoligotyping and multi-locus VNTR analysis (MLVA) are two molecular typing techniques routinely used to genetically characterize *M. bovis* isolates. Spoligotyping consist in the detection of presence or absence of the 43 spacers contained in the direct repeat (DR) locus [1, 25]. Spoligotypes are considered fairly stable, and are named according to an international nomenclature [1, 3]. Mycobacterial interspersed repetitive units–Variable numbers of tandem repeats (MIRU-VNTR) typing is based on a PCR amplification targeting several loci. Eight such loci are routinely used by the French National Reference Lab [1]: the resulting profiles are the numbers of repeats for each of the eight loci.

The combination of the two molecular techniques provides a good discriminating power (even if it is an order of magnitude lower than that of whole genome sequencing [26]) and the genotypes thus defined have been used in retrospective large scale epidemiological studies of the spread of *M. bovis* strains: more than 700 such genotypes have been identified among strains isolated in French cattle between 1997 and 2013 [1], of which 14 have also been observed in wildlife [16]. Genotypes that are significantly different from each other (i.e., a different spoligotype and/or a different VNTR profile at multiple loci), can be used to trace distinct epidemics.

In the present study, we focused on a study area located in Southwestern France (in the Landes and Pyrénées-Atlantiques departments), using data from 11 distinct *M. bovis* genotypes (this term will be used below to designate the combination of a spoligotype and a MIRU-VNTR profile) of which, according to surveillance data, nine affected cattle only (i.e. no infected animal was detected among badgers trapped in the vicinity of affected farms), and two others, simultaneously cattle and badgers. These genotypes differed from each other in spoligotype and/or MIRU-VNTR profile, for at least two of eight loci. Seven had never been reported in the study area before 2007, whereas four others had been reported in cattle at low incidence level. In total, 141 farms and 65 badgers were found infected between 2007 and 2019. In this area, apparent prevalence is low in wild boar populations (among wild boars harvested by hunting, 21/668 animals were found infected between 2012 and 2017, based on the analysis of pools of lymph nodes by culture and/or PCR detection) [16], and roe deer were not found infected. Red deer did not live in this area.

The objectives of this study were: (i) to reconstruct the 11 bTB epidemics in the study area between 2007 and 2019 and (ii) to analyze the respective roles of cattle and badgers in the transmission of *M. bovis* genotypes recovered in the two species, based on estimates of the intra- and inter-species reproduction numbers and generation times.

## Materials and methods

### Study area and epidemiological system

**Figure 2:**
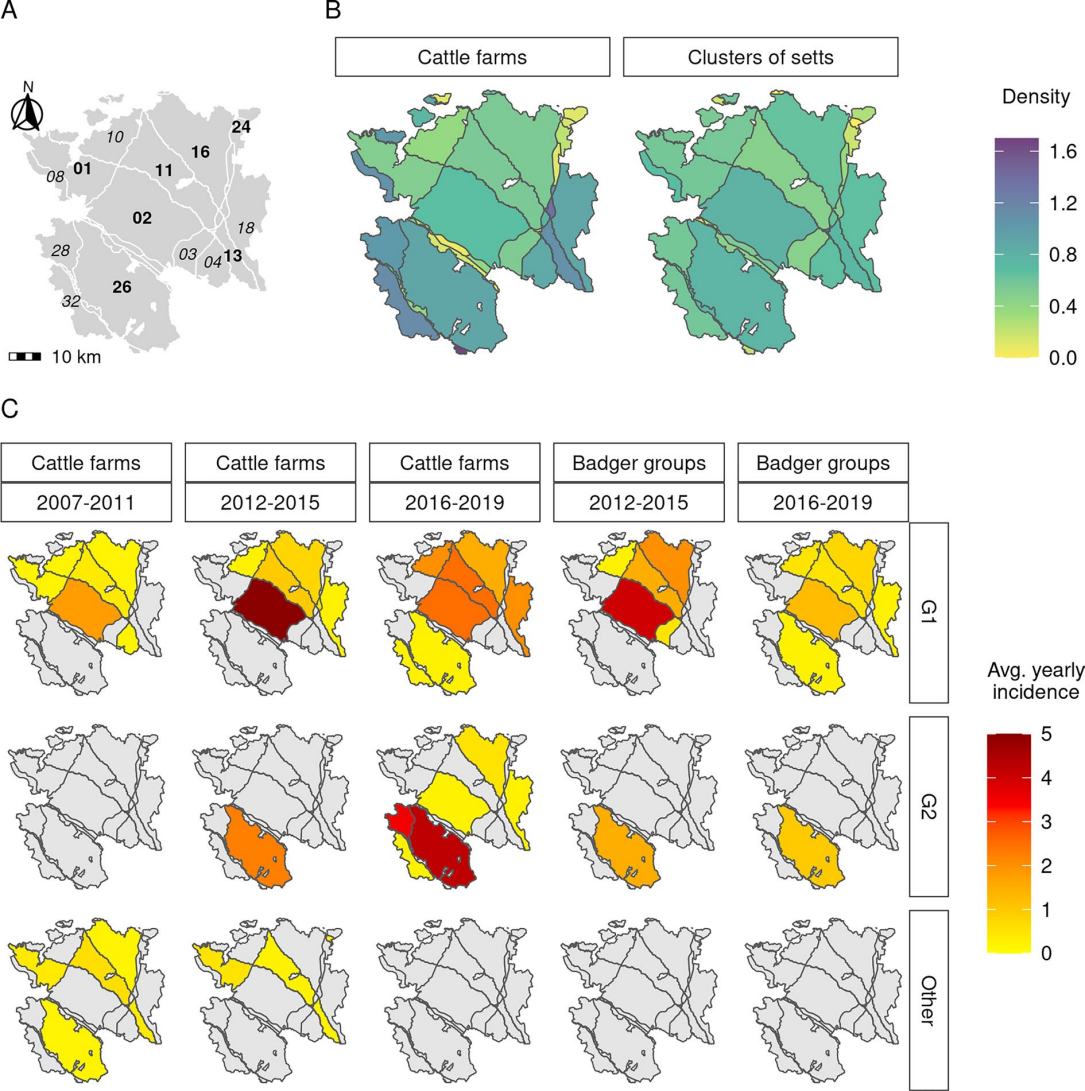
**Study area, population and bTB epidemiological data.** **A** Study area and subareas delimited by high traffic roads and rivers larger than 50 m (bold: subareas where the 11 genotypes of *M. bovis* were first detected, italic: other areas of > 30 km^2^). **B** Spatial density of farms and of clusters of badger setts (farms/clusters per km^2^). **C** Average yearly number of *M. bovis* detected infected farms and sett clusters in 2007–2011 (control protocol in farms based on total slaughter, no surveillance in badgers), 2012–2015 (reinforcement of surveillance in farms, beginning of surveillance in badgers), and in 2016–2019 (introduction of a test-and-cull protocol for bTB surveillance in farms).

The study period ranged from January 2007 to December 2019. The study area of 2735 km^2^ was located in Southwestern France, in two neighboring departments: Pyrénées-Atlantiques (PA) and Landes (Figure 1) to include all the municipalities (the smallest French administrative subdivision) where a census of badger setts had been performed between 2013 and 2015 (Figure 2A). Inside this area, we modeled an epidemiological system composed of two interacting metapopulations. The cattle metapopulation consisted of farms trading between each other and grazing on a set of pastures most of the year. We assumed trade and pasture neighborhood relationships supported *M. bovis* transmission between farms. The badger metapopulation was made of social groups using one or several neighboring setts, with a home range centered on these setts (see below). We assumed that badgers (especially young adults) could migrate between social groups, and that dispersal and contacts between groups at territory boundaries supported *M. bovis* transmission between social groups. As direct contacts between badgers and cattle have been shown to be very rare [27–32], we assumed that the inter-species transmission was only mediated by the environment, i.e. the pastures where both species were present and where *M. bovis* may survive for several months [33–36].

### Cattle-related data

We extracted cattle population data from two databases provided by the French Agriculture Ministry: farm sizes and cattle trade data were obtained from the French cattle tracing system (“Base de Données Nationale d’Identification”, BDNI) and pasture data (land parcels owned by farmers, used for cattle grazing) were obtained from the national graphic land survey (“Registre Parcellaire Graphique”, RPG) of 2013. Both datasets have been described previously [37]. They included 1946 farms with an average of 43.6 adult females and 8.0 pastures. The average farm density was 0.71 farm/km^2^.

The local animal health authorities provided the surveillance and control protocols implemented during the study period [37], which have evolved in three phases: 2007–2011, 2012–2015 and 2016–2019. Before 2012, bTB incidence was very low (Figure 2C) and cattle were screened using skin tests (Single Intradermal Comparative cervical Tuberculin Test [SICTT] in dairy farms, and Single Intradermal Tuberculin Test [SITT] in other farms) every two years or three years in Landes and in PA, respectively. Complete herd depopulation was applied to each breakdown farms until 2016 when a test-and-cull control protocol [38] was introduced in 40% of infected farms. From 2012, when infection was discovered in badgers and bTB incidence increased in cattle (Figure 2C), surveillance was reinforced by more frequent (annual) skin testing (using SICTT) in the municipalities of infected farms.

The bTB surveillance data were provided by the French Ministry of Agriculture: date and identifying number (ID) of the 151 detections of bTB in 141 farms (Figure 2C), with two detections in eight farms and three detections in one other farm. Molecular typing data (i.e. genotypes for each infected herd) were provided by the National Reference Laboratory (NRL, ANSES, Maisons-Alfort). In each infected farm, spoligotype was performed on all isolates, MIRU-VNTR on at least three isolates, or on all isolates if different MIRU-VNTR profiles were observed for a given spoligotype. Eleven *M. bovis* genotypes were detected in the study area during the study period (Table 1). Two genotypes were largely predominant, G1 (90 cases in 83 farms) and G2 (45 cases in 45 farms) (Table 2), mostly in the northern and southern part of the study area respectively (Figure 2C). Genotype G1 was already present in the study area before 2007, at a low level of incidence, unlike G2. Three of the nine other genotypes had also been reported in the study area before 2007 (Table 2).

**Table 1:** **Definition of the eleven genotypes identified in the study area, 2007–2019**.

Genotype	Spoligotype profiles		MLVA profiles (MIRU-VNTR)
	Name	Deleted spacers	
G1	SB0821	3, 5–6, 9, 16–21, 33, 39–43	6 5 5 3 11 2 5s^a^ 8
G2	SB0832	3, 5–6, 9, 16, 33, 39–43	6 5 5 3 11 2 4s 8
G3	SB0928	1–24, 26–28, 33, 39–43	6 7 3 3 10 2 7s 9
G4	SB0120	3, 9, 16, 39–43	5 3 5 3 11 2 5 6
G5	SB0121	3, 9, 16, 21, 39–43	5 2 5 3 8 2 5 6
G6	SB0120	3, 9, 16, 39–43	5 2 5 3 8 2 5 6
G7	SB0121	3, 9, 16, 21, 39–43	5 4 5 4 11 2 5 9
G8	SB0121	3, 9, 16, 21, 39–43	6 4 5 3 11 2 5 7
G9	SB0823	3, 5–6, 9, 16, 27–29	6 5 5 3 11 2 5s 6
G10	SB0851	3, 5–6, 9, 16, 33, 37, 39–43	5 5 5 3 11 2 5s 8
G11	SB0853	1–17, 23–24, 39–43	3 6 5 2 9 3 4 6

^a^The letter “s” denotes the presence of a truncated (“short”) repetition at a given locus.

**Table 2:** **Observed bTB detections in cattle farms and trapped badgers of the survey area, 2007–2019**.

Genotype	Cattle farms			Badgers	
	Detections (farms)	1st detection year (area^a^)	Last detection prior 2007	Animals (sett clusters)	1st detection year (area^a^)
G1	90 (83)	2007 (02, 11, 16)	2006	47 (44)	2012 (11)
G2	45 (45)	2012 (26)	None^*^	10 (9)	2013 (26)
G3	4 (4)	2007 (13)	2004		
G4	2 (2)	2009 (01)	2004		
G5	2 (1)	2012 (01)	None^*^		
G6	1 (1)	2007 (16)	None		
G7	1 (1)	2011 (26)	None		
G8	1 (1)	2012 (24)	None		
G9	1 (1)	2010 (11)	None^*^		
G10	1 (1)	2011 (11)	2001		
G11	1 (1)	2009 (11)	None^*^		
Untyped	2 (2)			8 (8)	
Total	151 (141^b^)			65 (60^c^)	

^a^The location of the different areas is given in Figure 1A

^b^One farm was detected infected by genotype G1 in 2008 and by genotype G9 in 2010; another farm was detected infected by genotypes G1 and G2 in 2018.

^c^One badger group was detected infected by genotype G1 in 2012 and by an untyped strain in 2016.

^*^Genotype never detected prior 2007 in the study area, but reported elsewhere in Southwestern France.

### Badger-related data

Badger population data consisted in 2668 setts identified by the census conducted between 2013 and 2015 (by hunters who were asked to actively prospect their communes of residence and to report each badger sett they found, without any specification of badger activity or type [39]). A badger density of 0.98 sett/km^2^ was measured. Major roads with heavy traffic and wide rivers (larger than 50 m) are natural barriers that hinder badger movements [40, 41]. We extracted the corresponding geographic data from the BD TOPO^®^ (2.1, IGN 2015) and BD Carthage^®^ (3.0, IGN 2014) databases and used it to split the study area into 41 subareas (Figure 2A) ranging from 1.45 to 500 km^2^. We assumed that badger movements and contacts could not occur between subareas. Social groups of badgers are known to use and maintain multiple setts in their territory, between which they range, in distances depending among other things on food resource availability or disturbance [42]. Field data collected in France showed that setts less than 500 m apart most often belong to a same social group [43]. We used this threshold to group setts into clusters, characterized by a maximal distance of 500 m between setts and assumed that these clusters included all setts used by a given social group. For each subarea, to build these clusters from the list of sett locations, we iteratively applied the following procedure:

(i) we defined a neighborhood network linking setts less than 500 m apart;
(ii) if the network consisted of isolated nodes, each sett formed a separate cluster and the procedure was stopped; otherwise we considered the largest component of the network (i.e. sets of nodes linked directly or indirectly) as a putative cluster of setts;
(iii) while the maximum distance between pairs of colonies in this putative cluster was > 500 m, we determined the most central sett in the putative cluster (the one with the largest number of neighbors in the network), and removed the sett that was furthest from it;
(iv) by construction, the remaining setts constituted a cluster, and we removed them from the list of sett locations before applying again steps (i)-(iv).

We obtained a total number of 1750 sett clusters, corresponding to a density of 0.64 sett clusters/km^2^ (Figure 2B), with a range of 1 to 8 ($\mu =1.5 ; \sigma =0.9$) setts per cluster. We then defined the home range associated to each cluster of setts (i.e. to each social group) based on (i) a Dirichlet tessellation around each cluster of setts, taking the closest sett to the centroid of all setts as the reference location for a cluster [42, 44]; and (ii) the intersection between each tile and a 1000 m buffer area drawn around this reference location, to obtain realistic home ranges [37]. Each home range was finally clipped using the outline of the subarea where the corresponding cluster of setts was located. In substantial subareas, i.e. wider than 30 km^2^ (*n* = 13), cluster density ranged from 0.47 to 0.78 cluster/km^2^ (while farm density ranged from 0.38 to 1.13 farms/km^2^) (Figure 2B).

In the study area, the *M. bovis* surveillance system (Sylvatub) in badgers started in 2012 [16] and included i) badger trapping (using stopped restraints placed near sett entrances –a capture method causing minimal injury [45]), with most of the badgers trapped in the municipalities of infected farms and ii) road killed badgers. All badgers trapped (and culled by headshot within traps, if necessary) or road killed were tested for *M. bovis* infection (culture and/or PCR detection on pools of lymph nodes with or without visible lesions) and associated with the nearest cluster of setts. The surveillance results (trapping or collection date, ID of the sett cluster, and infection status) were provided by Sylvatub (Figure 2C). The NRL provided the molecular typing results [25]. The two *M. bovis* genotypes detected in badgers were the two predominant genotypes in cattle: G1 in the northern part of the study area (47 infected animals from 44 clusters of setts), and G2 in the southern part of the study area (10 infected animals from 9 clusters of setts) (Table 2, Figure 2C).

### Model

We built a stochastic model operating in monthly time steps and embedding the two metapopulations of cattle farms and badger groups. We represented cattle farms and badger social groups by age-structured compartmental models, with three health states for cattle (S: susceptible, E: detectable by screening tests without detectable post-mortem lesions i.e. infected and non-infectious, and I: detectable by screening tests and presenting lesions detectable by post-mortem examination i.e. infected and infectious,) [46], and two for badgers (S: susceptible and I: infected and infectious). Indeed, Gormley and Corner pointed out the risk of *M. bovis* excretion by any infected badger, regardless of how advanced the infection was, with or without lesions [47]. We thus chose not to represent a latent state for badgers. Three age-classes were distinguished for badgers (cubs: < 1 year, sub-adults: 1–2 years, and adults: ≥ 2 years), and yearly age-classes were used for cattle.

**Figure 3:**
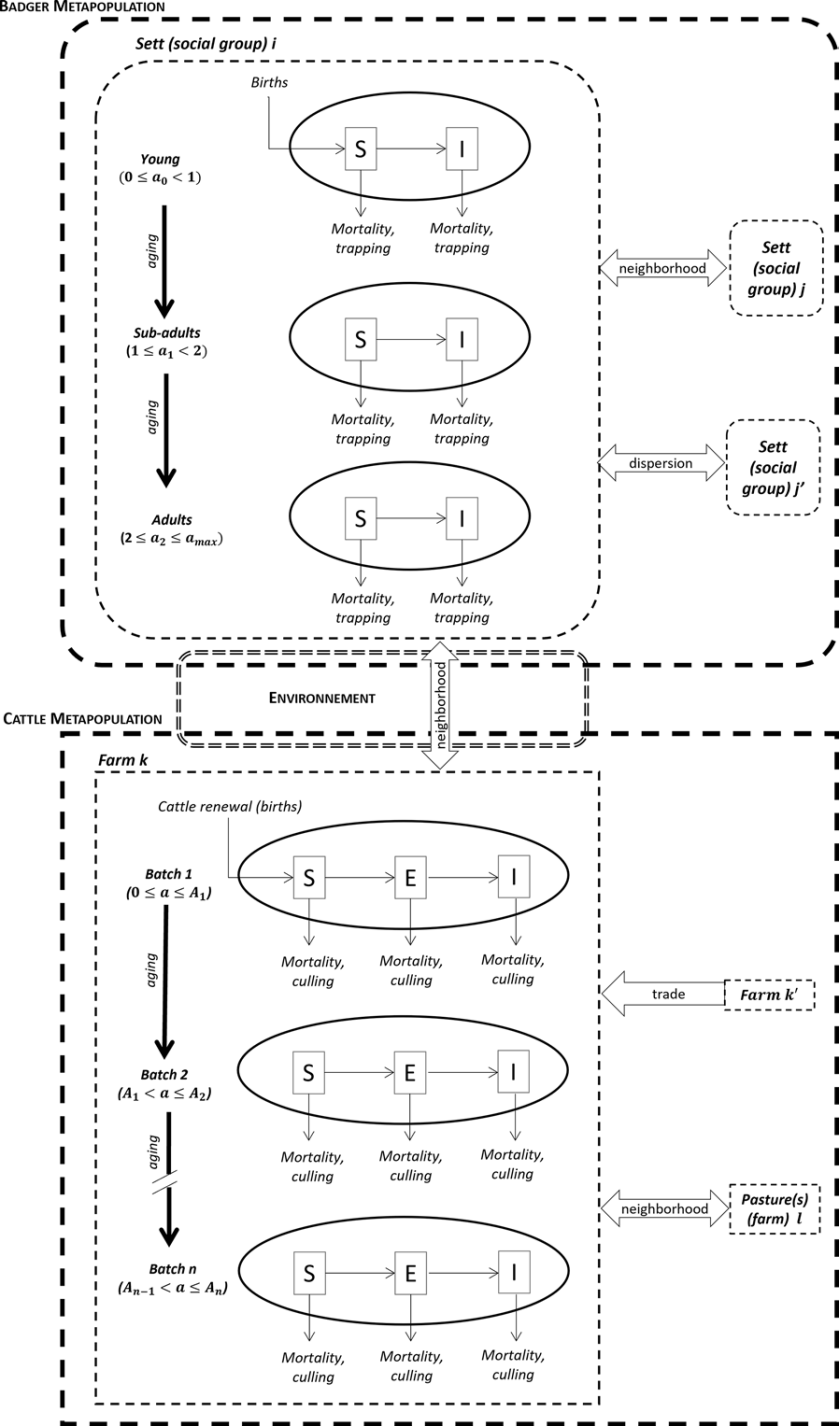
**Schema of the model of *M. bovis* transmission between badger social groups and cattle farms.** (S: susceptible; E: infected and non-infectious; I: Infected and infectious; Ai and Aj: ages limiting and characterizing batches of a cattle farm) (see Additional file 1 for details).

The model included three intraspecific transmission pathways, symmetric in both species (Figure 3, Additional file 1):

- Between individuals of the same population (badger social group or cattle farm): the transmission parameter was denoted ${\beta }_{W}^{B}$ for badger social groups, and ${\beta }_{W}^{C}$ for cattle farms (using the superscripts “B” for badger and “C” for cattle, and the subscript “W” for within-population),
- Between individuals of neighboring populations (based on neighborhood networks denoted ${\Phi }_{B-B}$ for badger groups, and ${\Phi }_{C-C}$ for cattle farms): we assumed that the transmission parameters (${\beta }_{N}^{B}$ for badger groups and ${\beta }_{N}^{C}$ for farms, the subscript “N” referring to neighboring populations) were proportional to the within-population transmission parameters, the ratios being denoted ${\varepsilon }_{N}^{B}$ and ${\varepsilon }_{N}^{C}$: ${\beta }_{N}^{B}={\varepsilon }_{N}^{B}$ ${\beta }_{W}^{B}$ and ${\beta }_{N}^{C}={\varepsilon }_{N}^{C}$ ${\beta }_{W}^{C}$,
- Between neighboring or distant populations, due to the movements of animals (dispersal of badgers between social groups, trade of cattle between farms): trade events were driven by data registered in the BDNI database. Badger dispersal was based on the neighborhood networks ${\Phi }_{B-B}$ (i.e. dispersal to the direct neighboring groups only), with an increasing dispersal probability when the number of subadults and adults exceeded a threshold (fixed by parameter *K*, see Additional files 1, 2). Perturbation effect was not represented as we assumed it was negligeable in our low badger density study area [48].

The interspecific transmission was assumed to be environment-mediated, according to a neighborhood network denoted ${\Phi }_{B-C}$ between cattle farms and badger social groups. Infectious animals were assumed to contaminate the pastures they ranged on (for cattle) or they visited (for badgers). The local survival of *M. bovis* in the environment allowed the subsequent infection of animals visiting the contaminated pasture (badgers) or placed on it (cattle). The transmission parameter from environment was denoted ${\beta }_{E}^{B}$, for badgers, and ${\beta }_{E}^{C}$ for cattle.

Since only one farm was found to be infected with > 1 genotype at the same time (Table 2) in the study area, we made the simplifying assumption that at a given time, a population could only be infected by a single genotype. Genotypes were defined by molecular markers considered stable and the probability of mutation from a genotype to another was assumed negligible. Under these two assumptions, a transmission tree represented the transmission of a unique genotype: that of the initially infected population on which the tree was rooted. We based the representation of a multi-type *M. bovis* epidemic upon the construction of transmission trees. Drawing the infector of each infected population (badger social group or cattle farm) during a simulation allowed to dynamically build transmission trees rooted on the population initially infected. A given population could however appear more than once in a transmission tree, or in several transmission trees, if it had been infected on several occasions.

### Parameterization

We extracted cattle population data (farm sizes and types) from the BDNI. Cattle-related parameters values are detailed in Additional file 1. We based the values of parameters that drive badger population dynamics on field observations or literature, except for three parameters that were calibrated based on recent field data: the threshold number of adult and sub-adult badgers in the sett cluster, below which mortality probability decreases, and above which dispersion probability increases, the dispersion rate, and the yearly probability of reproduction in a social group of at least two adults (Additional file 2). We simulated, using the calibrated model, 100 possible initial states of the entire badger metapopulation. The average number of badger groups was 1011 groups (range: 701–1329). The median density of animals was of 0.75 badgers per km^2^ (range: 0.45–1.02 badgers/km^2^) and varied between subareas, the highest in subareas 02 and 26 (0.96 and 0.89 badgers/km^2^, respectively), and the lowest in subarea 11 (0.53 badger/km^2^) (Figure 2, Additional file 3). Afterwards, at the beginning of each simulation, we randomly selected one of these 100 initial states to initialize the badger metapopulation.

The neighborhood network ${\Phi }_{B-B}$ between social groups was defined based on the immediate vicinity between home ranges (shared border). The neighborhood network ${\Phi }_{C-C}$ between farm pastures was built by defining as neighbors the farms whose pastures borders were less than 3 m apart (1.5 m being the geographic precision level of the dataset we used) [49]. We built the neighborhood network ${\Phi }_{B-C}$ between social groups and farms based upon the overlap of home ranges and pastures.

We used APMC-ABC (adaptative population Monte-Carlo approximate Bayesian computation) [50] to estimate five parameters driving the different *M. bovis* transmission pathways: between badgers of the same social group (${\beta }_{W}^{B}$), between badgers of neighboring social groups (${\varepsilon }_{N}^{B}$), from a pasture contaminated by infected cattle to the badgers visiting the pasture (${\beta }_{E}^{B}$), between cattle of neighboring farms (${\varepsilon }_{N}^{C}$), and from a pasture contaminated by infected badgers to the cattle using the pasture (${\beta }_{E}^{C}$). The prior distributions and summary statistics we used, as well as details of estimation procedure are given in Additional file 4.

The initial situation of *M. bovis* infection could not be determined with precision. We therefore made the conservative assumption that each of the 11 genotypes was already present in the study area at the beginning of the study period. Available data did not allow us to determine whether the different genotypes were originally present in badgers, cattle or both. Regarding the nine genotypes never reported in badgers and given the trapping measures in place since 2012, we assumed that they were initially absent from the badger population. Genotypes G1 and G2 may have been present in 2007 in one or both species, corresponding to nine possible initialization scenarios. We compared the nine scenarios using a model choice procedure specifically designed for inference using ABC, based on random forests classification methods [51] (Additional file 5). If the posterior probability (computed using the trained random forest) was low that the selected scenario corresponded to the true scenario, we analyzed the sensitivity of our results to the eight remaining scenarios. Because the incidence of bTB was significantly higher in our study area than in the surrounding areas during the study period, and because there were no reports of animal movements from other infected areas in France, we assumed that the risk of introduction of *M. bovis* during the study period could be neglected.

In cattle, infection was seeded at the beginning of each simulation, in one cow on farms where that type had been reported in the year of its first detection. This corresponded to a unique farm for the nine genotypes detected only in cattle and for G2, and to four farms for G1 (Table 2). In badgers, in the survey area (and more generally in France), *M. bovis* infection has always been detected in the vicinity of bovine outbreaks [16], we drew the initially infected social groups among the neighbors of the farms where the genotype was first reported (according to the ${\Phi }_{B-C}$ network), and infection was seeded in one adult badger.

### Model implementation, internal validation and exploitation

We evaluated the quality of fit by comparing, for the 22 summary statistics (Additional file 4, Table 2), the observed value with the predicted distribution in 10 000 simulation runs. We then performed an internal validation of the calibrated model by comparing the predicted and observed numbers of *M. bovis*-infected farms and social groups, stratified along the three dimensions of our model: time (13 years for farms and eight years for badger groups, as surveillance began in 2012 for this species), space (41 subareas) and genotype (11 types), yielding 9471 incidence values.

We used the transmission trees computed during each simulation to estimate the effective reproduction number (*R*) and the distribution of the generation time (*G*): a given transmission tree allowed computing, for each infected population, the average number of other populations it contaminated and their respective infection dates. Stratifying these calculations by couples of population types allowed obtaining four partial reproduction numbers and four generation time distributions: from cattle farms to badger groups (*R*_*CB*_*, G*_*CB*_), between farms (*R*_*CC*_*, G*_*CC*_), from badger groups to farms (*R*_*BC*_*, G*_*BC*_), and between badger groups (*R*_*BB*_*, G*_*BB*_). The overall reproduction number (*R*) was the dominant eigenvalue of the 2 × 2 matrix of partial reproduction numbers. We ran 10 000 simulations to estimate the reproduction numbers and generation times for farms and badger groups infected during each period (2007–2011, 2012–2015, 2015–2019), both globally, per genotype and per subarea. To avoid censoring, we ran these simulations until *M. bovis* was eliminated from all the populations that were infected by the 31^st^ of December 2019 and within 20 years (i.e. if not finished, simulations were stopped on the 31^st^ of December 2039). If there were any censored simulated data (i.e., populations infected before the 31st of December 2019 that were still infected 20 years later), they were discarded.

We computed the basic reproduction number at the beginning of the study period in the 13 subareas of > 30 km^2^. In each subarea we ran 200 simulated epidemics seeded in a single population (100 farms and 100 badger groups, randomly chosen), during which only transmission links from that population were allowed. Each simulation started on a randomly selected date of the year 2007. As above, to avoid censoring, we ran the simulations until *M. bovis* was eliminated from all populations that were infected by the 31st of December 2019. Simulated data therefore allowed computing the four partial basic reproduction numbers (*R0*_*CC*_, *R0*_*CB*_, *R0*_*BC*_, *R0*_*BB*_) as being the average number of transmission events for simulations seeded in farms (*R0*_*CC*_ and *R0*_*CB*_) and in badger groups (*R0*_*BC*_ and *R0*_*BB*_). The subarea-specific *R0* was the dominant eigenvalue of the 2 × 2 matrix of partial basic reproduction numbers. Confidence intervals were determined by bootstrap (1000 replicates).

A sensitivity analysis of the parameter estimates to three fixed parameters was finally performed: the duration of *M. bovis* survival on pastures (alternative values: 1.5 months and 6 months), the disease-induced mortality in badger (alternative value: 0.01 month^−1^), and the sensitivity of the diagnostic tests used for badger samples (alternative values: 0.65 and 0.85) (see Additional file 1 for the default values). Parameters were modified one at a time: we repeated the above parameter estimation procedure for the five resulting alternative parameterizations and compared the estimates and period-specific *R*s with those obtained using the default parameterization.

The model was coded in C +  + and operated as an R package using Rcpp version 1.0.7 [52]. We used R version 3.6.3 [53] to run simulations, the EasyABC package version 1.5 [54] for parameter estimation, and the abcrf package, version 1.8.1 [51], to compare *M. bovis* initialization scenarios in cattle and badger. The overall computation time was approximately 7 days (168 h) using 45 cores of a 64-cpu computer.

## Results

### Model calibration and internal validation

Comparison of initial infection scenarios in cattle and badgers led us to select the scenario in which G1 was initially present in four farms, and G2 both in one farm and one badger group: 22.8% of 1000 classification trees supported this scenario. Half as many classification trees (12.4%) supported the next scenario (G1 in four farms, G2 in one badger group). The least supported scenario combined the initial presence of G1 in one badger group and G2 in one farm (Additional file 5). However, the posterior probability that the selected scenario was superior to the eight others was low (0.25), we thus studied the eight remaining scenarios in the sensitivity analysis (see below).

Using the selected initialization scenario, the estimated transmission parameter from a contaminated pasture was much lower in cattle (${\beta }_{E}^{C}$, mean: 3.4 10^–5^ month^−1^ 95% CI: [1.4 10^–6^-1.7 10^–4^]) than in badgers (${\beta }_{E}^{B}$, mean: 0.04 month^−1^, 95% CI: [0.006–0.09]). Within badger social groups, the mean transmission parameter was estimated as 0.05 month^−1^ (${\beta }_{W}^{B}$, 95% CI [0.005–0.2]). We estimated each ratio of the transmission parameter between neighboring populations to within-population transmission parameter in cattle ${(\varepsilon }_{N}^{C})$ and in badgers ${(\varepsilon }_{N}^{B}$): the ratio ${\varepsilon }_{N}^{C}$ (mean: 0.009, 95% CI [0.0002–0.03] was slightly lower than ${\varepsilon }_{N}^{B}$ (mean: 0.02, 95% CI [2 10^–6^− 0.2]). However, for badgers, the credibility interval of ${\varepsilon }_{N}^{B}$ was large (and close to the bounds of the prior distribution: see Additional file 4), and the low influence of this parameter on the 22 summary statistics prevented us from estimating its value precisely. The model fit was satisfactory, as all the observed summary statistics were in the confidence interval of the predicted value (Additional file 4).

**Figure 4:**
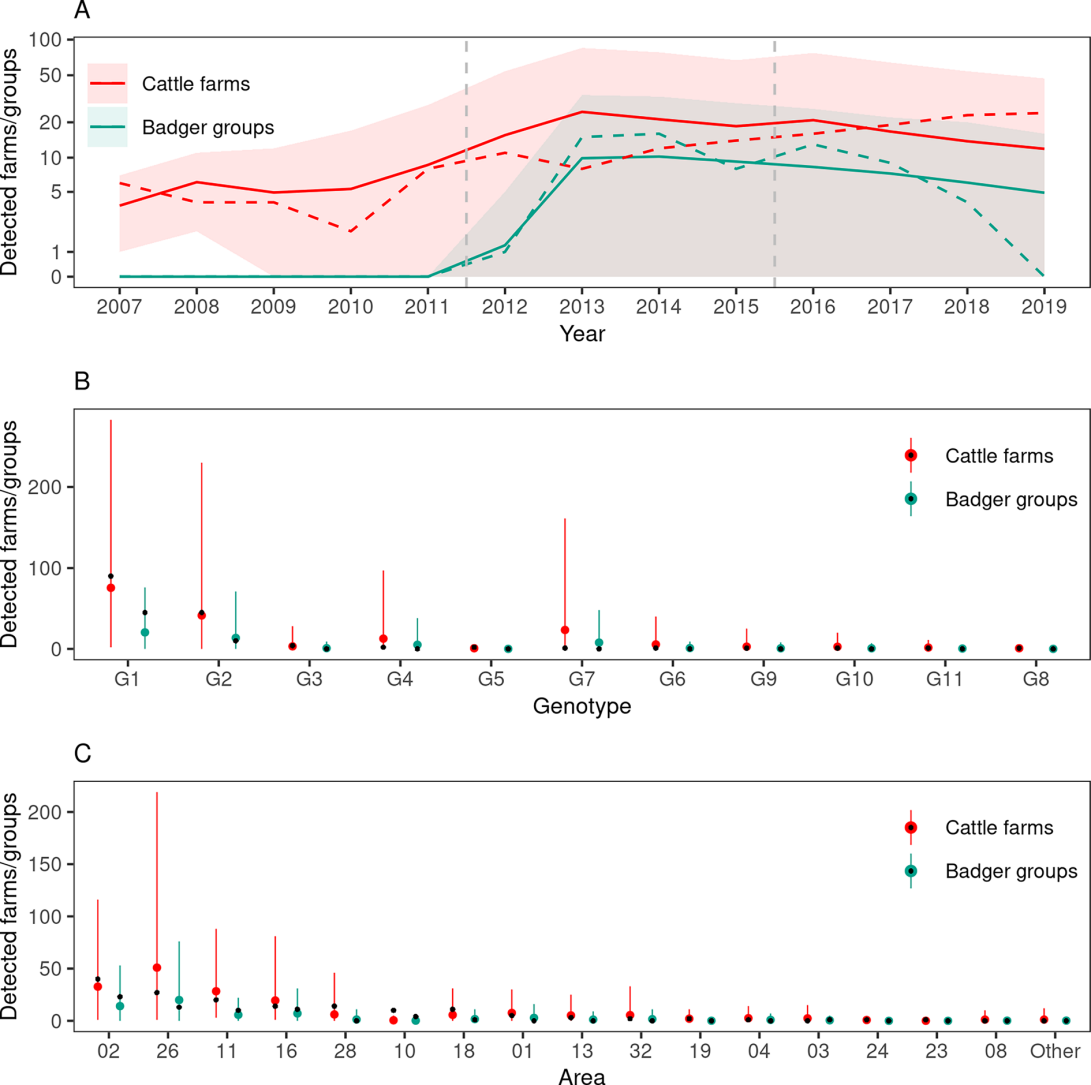
**Predicted and observed numbers of cattle farms and badger groups detected infected by *M. bovis***. Plain lines and colored dots: predicted values (average of 10 000 simulations), colored areas and vertical bars: 2.5% and 97.5% percentiles, dashed lines and black dots: observed values, vertical dashed lines: bounds of the three time periods. **A** numbers of detected infected populations by year, **B** by genotype, and **C** by area.

When stratified by species, time, space, and genotype, 17 of the 9471 observed apparent incidence values (0.2%) were outside the confidence interval of the predicted values. This corresponded to 0.6% of the 2905 strata in which the observed and/or mean predicted incidence was not zero. A graphical representation of the observed and predicted incidence in cattle and badger is given for each dimension of our model (time, genotype and space) in Figure 4, and for each pair of dimensions (space–time, space-genotype, and time-genotype) in Additional file 6.

### Reconstructed bTB incidence and prevalence in cattle farms and badger groups

**Figure 5:**
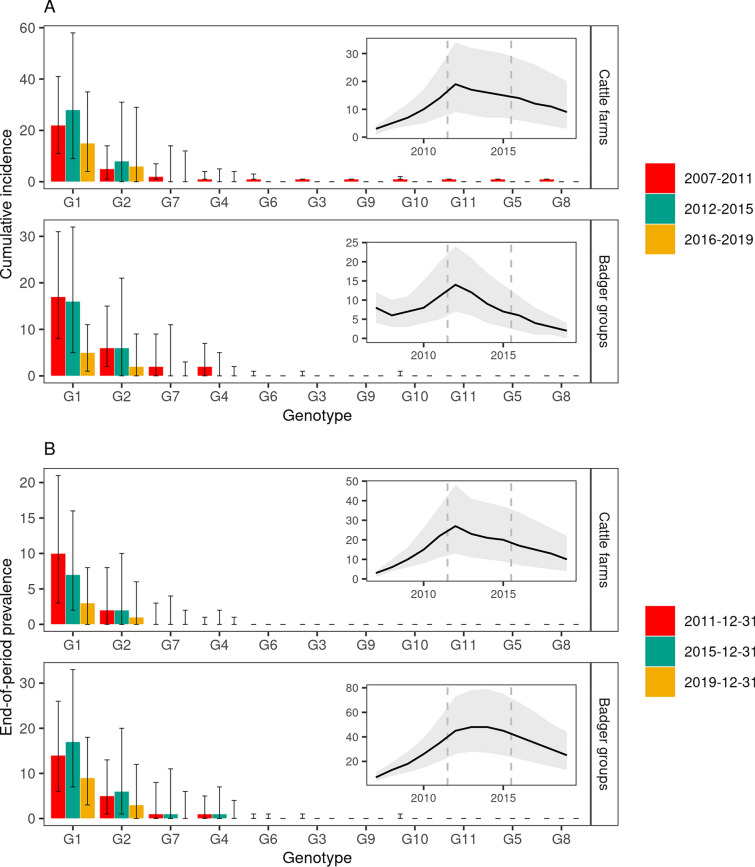
**Median predicted incidence and prevalence of bTB in cattle farms and badger groups.** **A** predicted cumulative incidence per period. **B** predicted end-of-period prevalence. Median values computed from 10 000 transmission trees, error bars: inter-quartile range. Insets: time evolution of the global incidence and prevalence, grey area: inter-quartile range, dashed lines: bounds of the three time periods.

The epidemics simulated using the calibrated model were very stable: bTB was still circulating at the end of the study period in the vast majority of simulations (99.4% of 10 000 simulations), both in cattle farms (91.3%) and badger groups (99.2%). Badger meta-population extinction never occurred during simulated epidemics, although trapping measures (i.e. badger culling) were predicted to have halved the occupancy rate of the sett clusters, from a median value of 0.59 in January 2007 (10 000 simulations, 95% CI 0.46–0.72) to 0.24 in December 2019 (95% CI 0.12–0.38). The reconstructed overall incidence peaked in 2012 in both species, with a median of 19 new infected farms and 14 new infected badger groups (Figure 5A, insets). While it decreased afterwards in badgers, with a median of two new infected groups during 2019, incidence remained at a higher level in cattle, with a median of nine newly infected farms in 2019 (Figure 5A, insets). The end-of-year median prevalence was similar to incidence in cattle farms, whereas it was markedly higher in badger groups (Figure 5). Indeed, once infected, the persistence of infection in badger groups (medians of 5.7, 3.2 and 2.9 years for groups infected in 2007–2011, 2012–2015 and 2016–2019, respectively) was much longer than in the cattle farms (medians of 1.5, 0.9 and 0.8 years for groups infected in 2007–2011, 2012–2015 and 2016–2019, respectively).

Computing genotype-specific incidence (Figure 5A) showed that, besides G2, already present in one farm and one badger group in the initial situation, the spillover from cattle farms to badger groups occurred in 95% of the 10 000 simulations for G1 (initially present in cattle farms only). This spillover occurred early in the simulations, as the median incidence was of 17 badger groups infected by G1 in 2007–2011. G1 and G2 were responsible for the majority of the reconstructed incidence and prevalence, with high median values in 2007–2011 and 2012–2015 followed by a decrease in 2016–2019. The predicted geographic distribution of the two genotypes was clearly different, and consistent with field observations: G1 being mostly prevalent in the northern part of the study area and G2 in its southern part (Figure 2 and Additional file 7). The spillover from cattle farms to badger groups was also predicted to occur for two other genotypes, G7 (60% of simulations) and G4 (58% of simulations), early in the study period but at a markedly lower rate than for G1: in both cases median incidence was of 2 badger groups in 2007–2011, and zero afterwards. The non-spillover observed for the 7 remaining genotypes was reproduced by the model, as the median incidence was always null for badger groups, and was of 1 farm in 2007–2011, and zero afterwards (Figure 5A).

### Analysis of transmission trees, effective reproduction numbers and generation times

**Figure 6:**
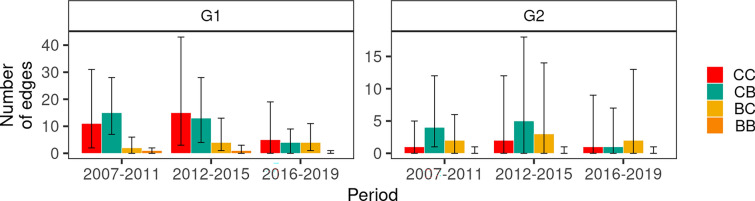
**Median number of edges in bTB transmission trees according to the transmission route.** CC: transmission between cattle farms. BB: transmission between badger groups. CB: transmission from cattle farms to badger groups. BS: transmission from badger groups to cattle farms. Median values computed from 10 000 transmission trees, error bars: inter-quartile range.

The 10 000 simulated transmission trees could contain 4 types of edges, according to transmission pathways: between badger groups, between cattle farms, from badger groups to cattle farms, and from cattle farms to badger groups (respectively denoted below BB, CC, BC and CB). Distribution of edges by type confirmed the early spillover of G1 from cattle to badgers, as CB edges were the most frequent in 2007–2011. The early back-transmission from badgers to cattle was rare: the CB to BC ratio was 7.5 in 2007–2011 (ratio of the median number of edges). Both directions of transmission occurred at similar levels in 2016–2019 (Figure 6). The CC edges were common in all three periods for G1, but not for G2: CB and BC edges were predominant, with CB edges more frequent than BC edges in 2007–2011 and 2012–2015, and the opposite in 2016–2019 (Figure 6).

**Figure 7:**
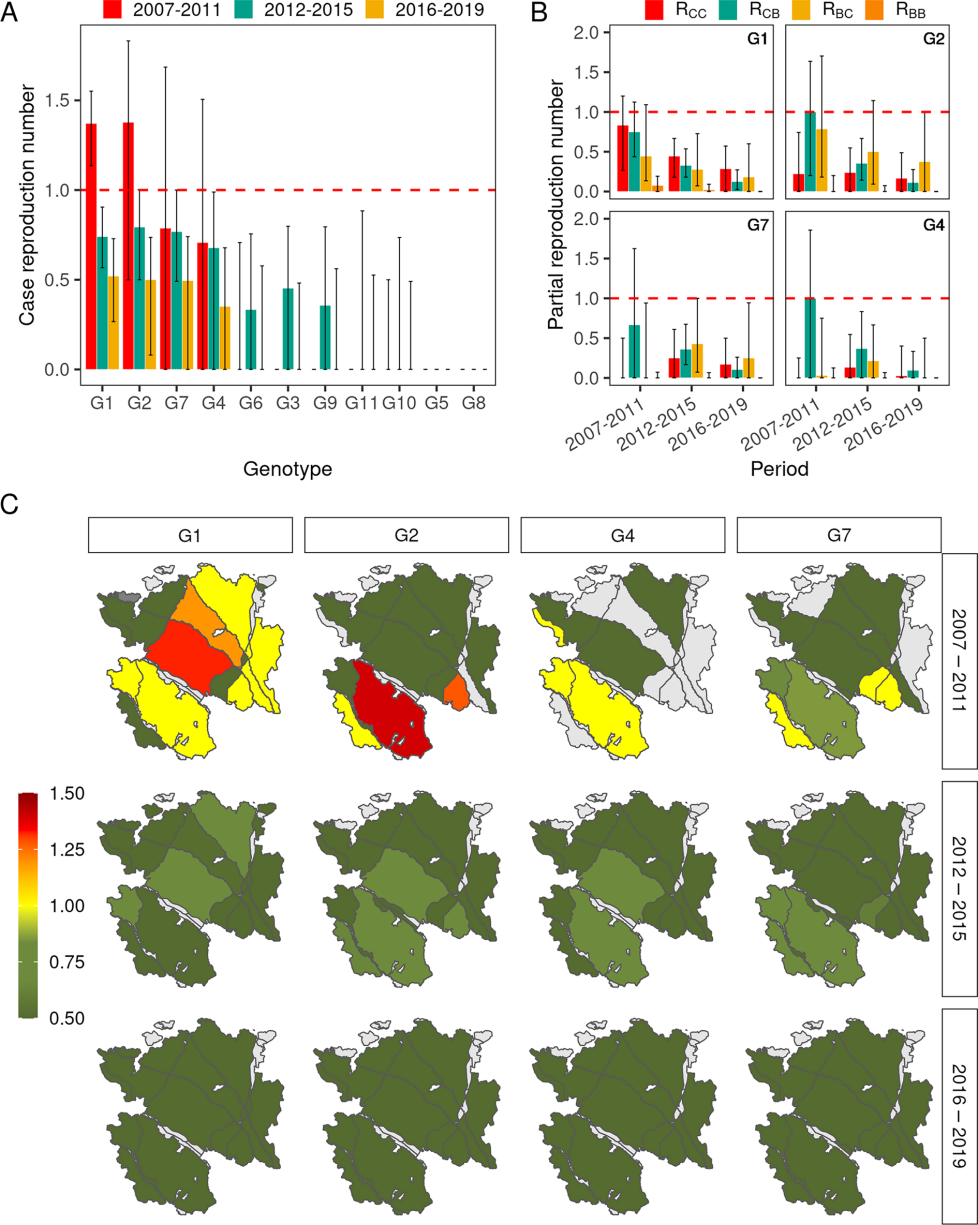
**Estimated reproduction number of bTB according to period, genotype and subarea.** **A** variations of the overall *R* according to the genotype and period. **B** variations of *R* according to the genotype, period and to the transmission route (*R*_*CC*_: between farms, *R*_*CB*_: from farms to badger groups, *R*_*BC*_: from badger groups to farms, and *R*_*BB*_: between badger groups. **C** variations of the overall *R* according to the subarea. Median values computed from 10 000 simulated transmission trees. Error bars: inter-quartile range.

The median overall effective reproduction number was 1.34 between epidemiological units (farms and badger groups) in 2007–2011 (inter-quartile range [IQR]: 1.14–1.55), 0.80 in 2012–2015 (IQR: 0.67–0.93) and 0.60 in 2016–2019 (IQR: 0.44–0.75). G1 and G2 were the two only genotypes for which the median *R* was > 1 for populations infected in 2007–2011: 1.37 for G1 and 1.38 for G2. During that same period, the median *R* was 0.79 for G7, 0.71 for G4, and zero for the seven remaining genotypes (Figure 7A). Regardless of genotype, the median R became < 1 for populations infected in 2012–2015, due to simulated control measures implemented (badger trapping) or strengthened (in farms) from 2012. The median *R* continued decreasing afterwards (Figure 7A). The geographic variations of *R* showed marked differences for populations infected in 2007–2011, with high median values in the southern part of the study area for G2 (especially subarea 26), and in its northern part for G1 (especially subareas 02 and 11). From 2012, the median *R* was < 1 in all subareas (Figure 7C).

None of the median partial *R*s was > 1, regardless of the period of infection and genotype (Figure 7B). In particular, for populations infected by G1 and G2 in 2007–2011, the within-species partial *R*s (i.e. *R*_*CC*_ and *R*_*BB*_) were always < 1 (the median *R*_*BB*_ being particularly low). Nevertheless, according to the model, farm-to-farm *M. bovis* transmission played an important role in the overall spread of G1 (especially in 2007–2011), contrary to G2, G7 and G4 for which *R*_*CC*_ was low and inter-species *R*s were predominant (Figure 7B).

In the simulated epidemics, farms infected by G1 transmitted *M. bovis* more rapidly to nearby badger social groups than to neighboring farms: in 2007–2011, the median generation time *G*_*CB*_ was 0.52 years, against 0.73 years for *G*_*CC*_ (Table 3). During that same period, badger groups transmitted *M. bovis* far more slowly: the median generation time *G*_*BB*_ was 1.31 years, and was lower than *G*_*BC*_: 2.41 years. Median generation time decreased during the subsequent periods, the contrasts between the types of edges being conserved: ${G}_{CB}<{G}_{CC}<{G}_{BB}<{G}_{BC}$. Conversely, generation time varied very little by genotype, with median values and inter-quartile ranges for G2, G7, and G4 being very close to those for G1 (Table 3).

**Table 3:** **Median generation time according to the genotype, the period, and the transmission route**.

Genotype	Period	*G*_*CC*_	*G*_*CB*_	*G*_*BC*_	*G*_*BB*_
G1	2007–2011	0.73 (0.63–0.85)	0.52 (0.44–0.61)	2.41 (1.75–3.17)	1.31 (0.81–2.00)
	2012–2015	0.66 (0.55–0.81)	0.49 (0.40–0.59)	1.87 (1.25–2.57)	0.92 (0.50–1.58)
	2016–2019	0.64 (0.51–0.87)	0.46 (0.35–0.59)	1.60 (0.95–2.35)	0.95 (0.46–1.75)
G2	2007–2011	0.75 (0.59–0.95)	0.52 (0.41–0.64)	2.31 (1.67–3.17)	1.19 (0.73–2.00)
	2012–2015	0.64 (0.50–0.79)	0.47 (0.38–0.57)	1.83 (1.25–2.43)	0.84 (0.47–1.42)
	2016–2019	0.63 (0.50–0.81)	0.46 (0.35–0.58)	1.58 (1.00–2.22)	0.83 (0.42–1.55)
G7	2007–2011	0.71 (0.50–0.92)	0.51 (0.39–0.63)	2.29 (1.62–3.10)	1.17 (0.67–1.96)
	2012–2015	0.63 (0.50–0.80)	0.46 (0.37–0.56)	1.74 (1.17–2.45)	0.83 (0.42–1.42)
	2016–2019	0.62 (0.50–0.82)	0.44 (0.33–0.58)	1.54 (0.92–2.24)	0.92 (0.42–1.75)
G4	2007–2011	0.75 (0.57–1.00)	0.48 (0.33–0.62)	2.50 (1.67–3.67)	1.42 (0.83–2.42)
	2012–2015	0.67 (0.51–0.87)	0.48 (0.37–0.62)	1.81 (1.12–2.62)	0.92 (0.50–1.50)
	2016–2019	0.62 (0.46–0.83)	0.45 (0.33–0.60)	1.56 (0.87–2.33)	0.83 (0.42–1.59)

Median values computed from 10 000 simulated transmission trees.

*G*_*CC*_ transmission between cattle farms, *G*_*BB*_ transmission between badger groups, *G*_*CB*_ transmission from cattle farms to badger groups, *G*_*BC*_ transmission from badger groups to cattle farms, Brackets: inter-quartile range

The basic reproduction number was significantly > 1 in four subareas (26, 28, 04 and 02, Table 4), suggesting a higher ability of *M. bovis* to invade the epidemiological system at these locations. Field data indicate that two of these four subareas corresponded to those where G1 and G2 were first detected and where the incidence was subsequently highest in cattle and badgers (subareas 02 and 26, Table 2). Additionally, *R0* was < 1 in eight subareas, among which three (01, 11 and 16) corresponded to subareas where six of the eleven genotypes we studied were reported in cattle with no subsequent spread (Tables 2 and 4).

**Table 4:** **Basic reproduction number at the beginning of the study period according to the subarea**.

Subarea^1^	*R0*^2^	*R0*_*CC*_^3^	*R0*_*CB*_^3^	*R0*_*BC*_^4^	*R0*_*BB*_^4^
26	1.53 (1.19–1.90)	0.71	1.45	0.64	0.41
28	1.51 (1.18–1.85)	0.74	0.87	1.09	0.27
04	1.50 (1.17–1.82)	0.67	1.30	0.71	0.38
02	1.40 (1.10–1.73)	0.59	1.20	0.74	0.31
13	1.10 (0.87–1.36)	0.50	0.58	0.92	0.21
03	0.98 (0.70–1.28)	0.61	0.96	0.31	0.19
18	0.95 (0.73–1.19)	0.38	0.54	0.70	0.28
32	0.92 (0.70–1.16)	0.48	0.38	0.69	0.33
16	0.91 (0.68–1.16)	0.41	0.82	0.42	0.23
11	0.88 (0.67–1.08)	0.60	0.48	0.42	0.15
01	0.72 (0.49–0.99)	0.45	0.65	0.27	0.08
08	0.64 (0.48–0.82)	0.31	0.46	0.37	0.13
10	0.60 (0.42–0.77)	0.24	0.61	0.21	0.25

^1^Subareas of > 30 km^2^, ranked by decreasing *R0*

^2^Maximal eigenvalue of the 2 × 2 matrix formed by *R0*_*CC*_ (transmission between farms), *R0*_*CB*_ (transmission from farms to badger groups), *R0*_*BC*_ (transmission from badger groups to farms), and *R0*_*BB*_ (transmission between badger groups). Brackets: bootstrap confidence interval (1000 replicates)

^3^Averaged over 100 simulations starting in a randomly chosen farm.

^4^Averaged over 100 simulations starting in a randomly chosen badger group.

### Sensitivity analysis

The eight alternative initial infection scenarios had very little impact on the posterior distributions of the five estimated parameters, and on the predicted values of reproduction numbers (Additional file 8). The trends described above for *R*, obtained using the selected scenario (G1 initially present in four farms, and G2 both in one farm and one badger group) were also obtained using the alternative scenarios: *R* > 1 in 2007–2011 and < 1 afterwards, intra-specific partial *R*s always < 1 with low values of *R*_*BB*_, high value of *R*_*BC*_ in any period. Alternative parameterizations of the disease-induced mortality (${\mu }_{d}$), of the sensitivity of diagnostic tests used in badgers (*Se*), and of the duration of *M. bovis* survival on pastures (*s*), did not significantly impact the the values and trends of the reproduction numbers, nor the posterior distributions of estimated parameters (although, concerning cattle, the median was increased by 24% when the survival duration was halved, whereas, for badgers, the median was decreased by 33% when the survival duration was doubled: both differences were small relative to the variance of the posterior distributions) (Additional file 8).

## Discussion

In this study, we analyzed the spread of 11 distinct *M. bovis* genotypes in a 2735 km2 area in Southwestern France between 2007 and 2019, using a spatially-explicit model of *M. bovis* transmission in badger and cattle metapopulations. Four genotypes were already present in cattle before 2007 (at a low incidence level), and there was no known wildlife infection prior to 2012. For nine of the 11 genotypes, the level of circulation remained very low, while two (G1 and G2) became widespread and were still circulating at the end of 2019. These differences in genotype spread were clearly related to the spillover from cattle farms to the local badger population, as G1 and G2 were reported in both cattle and badgers, whereas the nine other genotypes were only reported in cattle. The model we proposed was able to reproduce observed variations in incidence over space, time, and genotype. In particular, it correctly reproduced a spillover from cattle farms to the local badger populations for G1 and G2, and no spillover for seven other genotypes. For two genotypes (G7 and G4) the model predicted a very low dissemination in badgers (median prevalence of one infected social group), which is consistent with the absence of detection of these two genotypes in this species. Furthermore, the model was able to reproduce the contrasting diffusion of genotypes as, between 2007 and 2011, the median reproduction number was > 1 for G1 and G2, and < 1 for the nine remaining genotypes.

Analysis of intra-species and inter-species reproduction numbers showed that, in 2007–2011, the combination of cattle farms and badger groups formed a maintenance community for G1 and G2 (*R* > 1), and that neither the cattle farms nor the badger groups considered alone were maintenance populations (both *R*_*CC*_ and *R*_*BB*_ being < 1). A similar situation has been described for the Randomized Badger Culling Trial (RBCT) area, in the South West of England [23] and in Ireland [55]. Both studies are however difficult to compare, as the epidemiological units were not the same: populations in the present study (farms and badger groups), and animals in the RBCT [23]. This difference may explain the higher values of inter-species reproduction numbers in our study (ranging 0.4–1.0 for G1 and G2, whereas they were < 0.05 in the RBCT [23]), and lower values for *R*_*BB*_, the badger-to-badger reproduction number (< 0.08 in our study, whereas RBCT data were best explained by a value close to 1 [23]). In addition to the differences in epidemiological units, the lower *R*_*BB*_ we obtained might be explained by the lower density of badgers in our study area (see Additional file 3: 0.96 (0.58–1.36) maximum median number of badgers per km^2^, compared to the RBCT: 4.9 to 9.8 badgers per km^2^ [56]), and were consistent with the low level of between-group dispersion demonstrated by Jacquier et al. [57], based on the genetic structure of badger social groups in France. The low level of *R*_*BB*_ is also consistent with a recent phylodynamic analysis of data from Northern Ireland [58]: comparison of several models of *M. bovis* reservoirs led the authors to select a model where a reservoir population (which may be a badger social group) would be able to hold infection at a local scale, even without *M. bovis* circulation in cattle, and to transmit the bacteria back to cattle farms, thanks to regular interactions.

Although combining spoligotyping with MIRU-VNTR allows obtaining a good discriminating power when genotyping *M. bovis* isolates, it is an order of magnitude lower than that provided by whole genome sequencing (WGS). The fact that a given lineage of *M. bovis* could have changed genotype during the study period would have biased our analyses. This could have been detected using WGS, but not with the genotyping methods we used. However, because they differed strongly from each other (i.e. by spoligotype or, when the spoligotype was identical, by two to four loci of the MIRU-VNTR profile) this risk seemed negligible. Besides, the use of WGS might have allowed us to identify distinct lineages within a given genotype, which would have led us to increase the number of initially infected epidemiological units (i.e. the number of lineages initially present in the area). Calculated reproduction numbers would then have been lower, and our estimates may thus have been over-estimated. Moreover, we assumed that at a given time step, only one genotype was circulating in an infected cattle farm or social group of infected badgers. If incorrect, this assumption could have led us to underestimate reproduction numbers. However, each trapped badger was culled, analyzed and, if infected, its isolate was genotyped. This protocol never allowed detecting more than one genotype in infected groups during the study period. In infected farms, all infected animals were genotyped, and only one farm was found infected by more than one genotype. This suggests that, if our hypothesis was incorrect, the underestimation of reproduction numbers was probably low.

Epidemiological data suggest that, in our study area, spillover from cattle to badger groups mainly occurred in two distinct areas for the two genotypes implicated: subarea 26 for G2, and subarea 02 for G1 (Figure 2). Badger densities in these subareas was the highest of the whole study area (0.90 and 0.97 badger/km^2^ for subareas 26 and 02, respectively, see Additional file 3), similar to values reported from the island of Ireland (0.82–1.06 badger/km^2^ in [59]), and *R0* was significantly > 1 (Table 4). In addition, we found *R0*s < 1 in the five areas where the nine genotypes other than G1 and G2 were first reported, apart for G7 first reported in subarea 26, like G2 (Tables 2 and 4). These results suggest that the contact networks we modeled satisfactorily reproduced field conditions that may favor (or penalize) local spread of *M. bovis* upon introduction (and its spillover to badgers). Indeed a previous study has shown that landscape and environmental variables (e.g. the percentage of sand in soil composition 500 m around setts, or the number of crop parcels 1000 m around setts) were associated, at a very local scale, with the *M. bovis* concomitant infection in both cattle and badgers [39]. The impact of local conditions on the spread of *M. bovis* has also been underlined by Birch et al. [60] for high incidence areas in England, where the exposure of a minority of farms (25%) to a local environmental reservoir of *M. bovis* (which may correspond to badger social groups) allowed to closely reproduce the spatial spread of bTB.

In addition to spatial aspects, the analysis of generation time distributions gives indications of how, according to the model, the different transmission pathways were organized in time in our study area. Our results suggest that a cattle farm infected in 2007–2011 transmitted *M. bovis* as quickly (or slightly faster) to neighboring badger social groups (*G*_*CB*_) as to neighboring cattle farms (*G*_*CC*_). In contrast, infected badger groups transmitted *M. bovis* to neighboring badger groups (*G*_*BB*_) and to cattle farms (*G*_*BC*_) after a long period of time, especially in the latter case (> 2 years). In our study area, the (relatively) fast transmission between cattle farms would have allowed *M. bovis* to first colonize areas where the spatial organization of pastures and badger home ranges, as well as the landscape [39], favored inter-specific contacts, and where the spillover to local badger social groups would have also been (relatively) rapid. Infected badger groups would then have become the site of prolonged circulation of *M. bovis* (over several years) and transmit the pathogen to cattle farms in a second phase, possibly after the initial spread between farms has been controlled. This mechanism could also help explain the recrudescence of infection observed in some farms. Recently, Rossi et al. [61] combined mathematical modelling and phylodynamics to analyze the bTB emergence in a low-risk area of north-west England with no reported infection in wildlife. Their study clearly showed the importance of early amplification of *M. bovis* by transmission between cattle farms for the establishment of bTB in the local badger population, which subsequently retransmit *M. bovis* to cattle.

A consequence of this two-step dynamic of *M. bovis* transmission is that inter-species transmission would first occur predominantly from cattle to badger, and later in the opposite direction. This is consistent with the transmission trees we simulated, where the ratio of the number of transmission edges from cattle to badger (CB) to the number of transmission edges from badger to cattle (BC) was initially > 1, and became ≤ 1 afterwards. Although the epidemiological units are different, the CB/BC ratios we obtained can be qualitatively compared with those obtained in phylodynamic studies, which were > 1 in some studies [61, 62], and < 1 in other [63]. In England, Tonder et al. recently analyzed retrospectively 12 putative transmission clusters reported between 1999 and 2008, and found CB/BC ratios > 1 in four clusters, and < 1 in the eight other [64]. In our study area, Duault et al. [65] found a CB/BC ratio < 1. This diversity in the CB/BC ratio probably reflects differences in the same epidemiological systems over time. However, if as suggested by our results, this ratio is not constant over time, its estimation could be biased by a non-uniform distribution of the isolation dates of the sampled strains, relative to the temporal evolution of the epidemic. The assumption of constant migration rates in the phylodynamic models used may also not be appropriate.

Wild boars were not considered in our study, although a few *M. bovis*-infected animals have been reported in the study area. Contrary to Mediterranean Spain where wild boar populations act as reservoirs of *M. bovis* [13], the wild boar is considered in France as a spillover host, which reveals the presence of *M. bovis* in wildlife and is able to disperse the bacterium over long distances [15, 17, 66, 67]. In our study area, the ability of the model to correctly reproduce incidence data based on the badger-cattle system alone suggests that wild boars played a minor role in the spread of the disease. This role (especially the spatial dispersion) remains however to be evaluated. In addition, adverse epidemiological outcomes attributed to movements of badgers after culling campaigns have been described in England [68]. Such indirect effects of trapping measures were not included in our model, as field data did not suggest their existence, probably due to a much lower badger density and culling pressure in our study area. Allen et al. work in Ireland seems to point in this direction [48], but this conservative hypothesis needs to be confirmed in the future in our study area, despite a similar badger density context.

Bovine tuberculosis control measures, first implemented in the 1950s and targeted at cattle farms, have proven effective in causing a significant decrease in incidence in European countries (from 13.5% in 1965 to < 0.1% in 2000, in France [69]) and have led to the eradication of bovine tuberculosis in many European regions. However, eradication has not been achieved in areas where *M. bovis* circulates in multi-host systems. A recent estimate [7] suggests that the total number of infected animals is of the same order of magnitude in cattle and badgers in central and western Europe. Where *M. bovis* circulates in a badger-cattle system similar to the one we studied (i.e. with a low to moderate badger density and a cattle-badger interface comparable to our study area), our results suggest that eradication is possible, since control measures allow to lower *R* below 1. The weight of interspecies transmission in the overall dynamics suggests that long term control protocol in farms and biosecurity measures are essential, especially on infected farms or with a history of bTB. Finally, even if *R* < 1, bTB eradication is a long-term prospect because of the prolonged infection of social groups of badgers. Vaccination of badgers around bTB-infected farms is a promising tool (with evidence suggesting that badger vaccination is not inferior to badger culling [70] as a control policy for cattle herds) that could reduce the time of infection in badger populations, by limiting the intra and interspecies *M. bovis* transmission and our model could be adapted to help evaluate its protective efficiency at population level.

## Supplementary Information

**Additional file 1: Model description.**

**Additional file 2: Calibration of the parameters of badger population dynamics.**

**Additional file 3: Density of cattle and reconstructed badger density, at the beginning of the study period, in subareas wider than 30 km2.**

**Additional file 4: Estimation of M. bovis transmission parameters.**

**Additional file 5: Choice of the bTB initialization scenario.**

**Additional file 6: Model fit to observed data along the three dimensions of the model: genotype, spaceand time period.**

**Additional file 7: Geographic variations of the reconstructed incidence and prevalence.**

**Additional file 8: Sensitivity analysis.**

## Data Availability

The population and epidemiological data used in this work are the property of the French Ministry of Agriculture, from which they can be requested. Geographic data are in the public domain and can be obtained from IGN [77].
