# Supplementary material for: Analysis of a multi-type resurgence of Mycobacterium bovis in cattle and badgers in Southwest France, 2007-2019
Source: Vet Res. 2023 May 3;54:41. doi: 10.1186/s13567-023-01168-8 (PMC10158257; doi:10.1186/s13567-023-01168-8)
Supplement: Supplementary file 1 — Additional file 1: Model description. [file 13567_2023_1168_MOESM1_ESM.docx]

# Additional file 1. Model description

The stochastic model embedded two metapopulations (cattle farms and badger groups), in which we modeled populations in a symmetrical way, incorporating three processes: demography, infectious processes, and bTB control (Figure 1). The within-farm submodel was adapted from [46] where a detailed description can be found.

**Demographic process**

*Badger groups*

Male and female badgers were represented in the model, and we distinguished three age classes: cubs (*a_0_*: 0-1 years old), subadults (*a_1_*: 1-2 years old) and adults (*a_2_*: >2 years old). Only the adults participated in the reproduction, giving birth to a litter in March, when there was at least 2 adults in the group, with a probability $\rho$ (Table 1). The size of the litter ranged from 1 to 5 cubs and was randomly sampled in a discrete distribution $\eta$ based on field observations (Table 1). Natural mortality could occur yearlong, with a monthly probability depending on the age class and on number of adults and subadults in the group: $\mu_{a}(N_{a1}+N_{a2})/K$ (with $(N_{a1}+N_{a2})/K$ truncated on the interval [0-1]), where $\mu_{a}$ was the maximal probability of mortality for age class a, *N_a_* the number of animals of age class *a*, and *K* the threshold on the total number of adults and subadults below which the probability of mortality decreases and above which the dispersion probability increases.

Dispersion of subadults and adults to the immediate (i.e. contiguous) neighboring setts occurred randomly from March to November. We assumed this probability was zero when the number adults and subadults was ≤2 (i.e. a breeding pair), and increased when the group size was larger. This probability was: $\delta(N_{a1}+N_{a2}-2)/K$ (truncated on the interval [0-1]), where $\delta$ is the probability of a dispersion event (Table 1). In the case of dispersal, we assumed that the animal would, if possible, go to a group with a single adult or subadult (in order to form a breeding pair), that it would otherwise go to an empty group, and that in other cases it would choose its destination at random among the remaining neighboring groups (i.e. short distance dispersion and no super-ranging behavior).

**Figure 1. Schematic representation of the model of *M. bovis* transmission between badger social groups and cattle farms**.


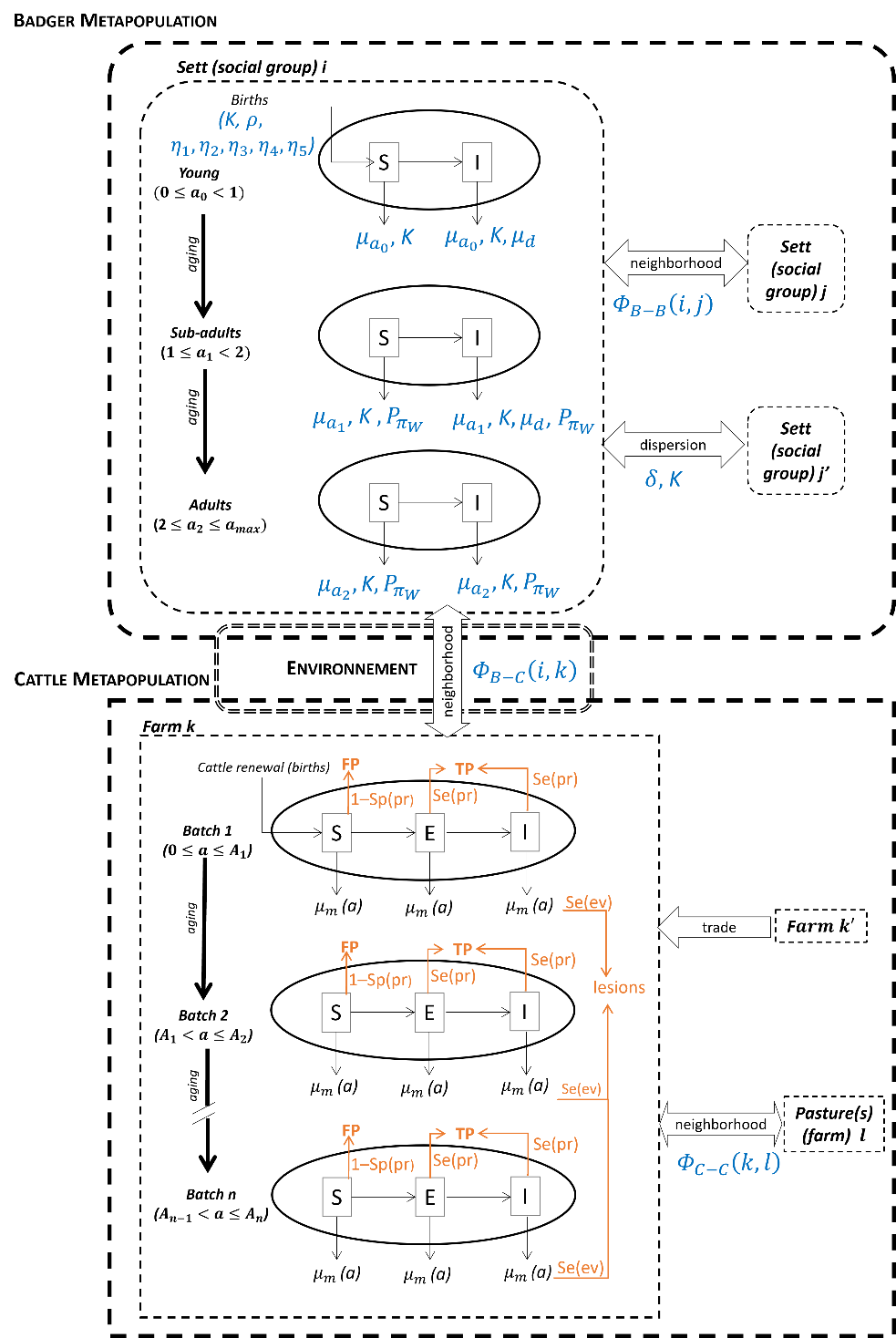


Black arrows represent demographic and infection dynamics, orange arrows the cattle active and passive surveillance protocols. Blue text corresponds to model parameters*.* *K*: threshold on the total number of adults and subadults, below which mortality probability decreases, and above which dispersion probability increases. *ρ*: probability of reproduction. $\mu_{a}$: natural mortality for age-class *a_._* *δ*: dispersion parameter. $\pi_{x}$: monthly removal probability in commune *x*. 𝛷*_B_*_−_*_B_*: adjacency matrix of the neighborhood network between badger groups. 𝛷*_B_*_−_*_C_* _:_ adjacency matrix of the neighborhood network between badger groups and farms. 𝛷*_C_*_−_*_C_*: adjacency matrix of the neighboring network between farms. $\mu_{m}(a)$: culling rate for month *m* and age-class *a*. Se(pr), Sp(pr): screening-test sensitivity and specificity. FP: false positive; TP: true positive. Se(ev): sensitivity of routine slaughterhouse examination of carcasses.

**Table 1. Model parameters for the badger metapopulation.**

| **Notation** | **Description** | **Value** | **Source** |
| --- | --- | --- | --- |
| *K* | Threshold on the total number of adults and subadults, below which mortality probability decreases, and above which dispersion probability increase | 0.90^a^ animals [0.79–0.98]^b^ | Calibration |
| $\rho$ | Probability of reproduction | 0.40^a^ [0.33–0.51]^a^ | Calibration |
| $\eta_{1}$ | Litter size probability: 1 cub | 0.27 | Field data^c^ |
| $\eta_{2}$ | Litter size probability: 2 cubs | 0.45 | Field data^c^ |
| $\eta_{3}$ | Litter size probability: 3 cubs | 0.18 | Field data^c^ |
| $\eta_{4}$ | Litter size probability: 4 cubs | 0.05 | Field data^c^ |
| $\eta_{5}$ | Litter size probability: 5 cubs | 0.05 | Field data^c^ |
| $\mu_{a_{0}}$ | Mortality parameter for cubs | 0.02 month^-1^ | [71] |
| $\mu_{a_{1}}$ | Mortality parameter for sub-adults | 0.005 month^-1^ | [71] |
| $\mu_{a_{2}}$ | Mortality parameter for adults | 0.005 month^-1^ | [71] |
| *δ* | Dispersion parameter | 0.01^a^ month^-1^ [0.002–0.02]^b^ | Calibration |
| $\mu_{d}$ | Disease induced mortality parameter | 0 | [72], field data^d^ |
| $s$ | Duration of *M. bovis* survival on pastures | 3 months | [33, 34] |
| $Se$ | Sensitivity of diagnostic tests used in wildlife | 0.75 | [16] |
| $\pi_{A}$ | Monthly removal probability for sub-adults and adults whose sett cluster is in category A municipalities^e^ | 0.05^a^ month^-1^ [0.03–0.08]^b^ | Calibration (Additional file 2) |
| $\pi_{B}$ | Monthly removal probability for sub-adults and adults whose sett cluster is in category B municipalities^f^ | 0.04^a^ month^-1^ [0.03–0.06]^b^ | Calibration (Additional file 2) |
| $\pi_{C}$ | Monthly removal probability for sub-adults and adults whose sett cluster belongs to a municipality neither A nor B | 0.005^a^ month^-1^ [0.004–0.007]^b^ | Calibration (Additional file 2) |
| $\beta_{W}^{B}$ | Transmission parameter between badgers of the same group | estimated |  |
| $\varepsilon_{N}^{B}$ | Proportion of within-group FOI exerted on animals of neighbouring groups | estimated |  |
| $\beta_{E}^{B}$ | Transmission parameter from a contaminated pasture | estimated |  |

^a^Mean value of the posterior distribution. ^b^95% posterior credibility interval. ^c^Mickaël Jacquier, results of the analysis of data collected using camera traps, personal communication. ^d^No pattern of severe lesions was observed in badgers necropsied in the study area. ^e^Municipalities with at least one cattle detected outbreak. ^f^No cattle outbreak detected in the municipality, but neighbouring municipalities with cattle outbreaks.

*Cattle farms*

Only females were represented in the model, since in most farms, males are raised separately from females and slaughtered young, and do not play a significant role in *M. bovis* transmission [35, 36]. Herd size was assumed constant and fixed, for each farm, to the values obtained from the cattle tracing system (BDNI) on 2013-08-01 (i.e. in the middle of the study period, during the grazing period). Culling (and natural mortality) could occur yearlong, with culling rates depending on farm type, which were estimated from BDNI data (Table 2). To keep herd size constant, culled animals were replaced by healthy heifers of the 1^st^ age class.

Animals were grouped in batches, depending on their age (yearly age classes with a maximum age of 15 years) and on the farm type. We distinguished three farm types, based on a simplified version of the typology proposed by Sala et al. [73]: dairy and mixed herds (>10 cows of a dairy or mixed breed: 324 farms having an average of 76.2 cows), beef herds (>10 cows of a beef breed: 922 farms having an average of 54.6 cows), and other herds (700 farms having an average of 14.1 cows). Four groups of animals were considered for dairy and mixed herds (cows, calves, 1-year-olf heifers and 2-years-olf heifers, the 3 latter being merged in winter), three for beef herds (cows with their calves, 1-year-olf heifers and 2-years-olf heifers), and a single group for the other herds.

Model equations describing the demographic process in farms can be found in [46].

**Infectious process**

*Badger groups*

Two health states were distinguished for badgers: susceptible (*S*) and infected (*I*). At a given time step, the force of infection (FOI) exerted on a badger of age class *a* in group *i* was defined by the following formula, in which the letter *B* denotes badgers:

1. $\lambda_{i,a}^{B}\left( t \right)= \lambda_{i}^{B, W}\left( t \right)+ 1_{a}^{N}\left( t \right)\sum_{j\mathcal{\in B}} \Phi_{B-B}\left( i,j \right)\lambda_{j}^{B, N}\left( t \right)+ 1_{a}^{E}\left( t \right)\sum_{k\mathcal{\in F}} \Phi_{B-C}\left( i,k \right) \lambda_{k}^{B,E}\left( t \right)$

where:

- $\lambda_{i}^{B, W}\left( t \right)$ is the frequency-dependent within-group FOI in group *i*, with transmission parameter $\beta_{W}^{B}$;
- $\lambda_{j}^{B, N}\left( t \right)$ is the FOI exerted by group *j* on the neighboring groups, with a transmission parameter assumed proportional to the within-population transmission parameter ($\beta_{W}^{B}$), the proportionality factor being $\varepsilon_{N}^{B}$;
- $\lambda_{k}^{B,E}\left( t \right)$ is the environment-mediated FOI exerted by pastures of farm *k* on badgers visiting it, with transmission parameter $\beta_{E}^{B}$: $\lambda_{k}^{B,E}\left( t \right)=\beta_{E}^{B}$ if environment is contaminated and zero otherwise;
- $\mathcal{B}$ is the set of badger social groups and $\mathcal{F}$ the set of farms;
- $1_{a}^{N}\left( t \right)$ and $1_{a}^{E}\left( t \right)$ are indicator functions respectively representing the ability of badgers of age *a* to have contacts with neighboring social groups or to visit pastures (March-November for adults and subadults, May-November for cubs);
- $\Phi_{B-C}\left( i,k \right)$ is the adjacency matrix of the neighborhood network between badger groups *i* and cattle farms *k* (1 if shared environment, 0 otherwise);
- $\Phi_{B-B}\left( i,j \right)$ is the adjacency matrix of the neighborhood network between badger groups *i* and *j* (1 if neighbors, 0 otherwise).

Environmental contamination was only considered for interspecies transmission: a pasture was assumed to be contaminated by *M. bovis* as soon as infectious cattle were present (i.e. the within-group FOI of cattle was non-null), and remained so during *s* months (Table 1).

The number of new infected badgers of age *a,* in social group *i*, during time step *t*, was then computed as follows:

1. $n_{i,a}^{SI}\left( t \right)=Binom\left( B_{i,a}^{S}\left( t \right), 1-e^{-\lambda_{i,a}^{B}\left( t \right)} \right)+\sum_{j\mathcal{\in B}} Bern\left( \Phi_{B-B}\left( i,j \right) 1_{i,j,a}^{Disp}\left( t \right)\frac{B_{j,a}^{I}\left( t \right)}{B_{j}(t)} \right)$

where the 1^st^ term represents the infection of healthy badgers already belonging to the social group and the 2^nd^ term the dispersal of infected badgers from the neighboring social groups:

- $B_{i,a}^{h}\left( t \right)$ denotes the number of badgers in health state *h*, in social group *i*, of age *a*;
- $B_{j}(t)$ the total number of badgers in social group *j* at time *t*;
- $1_{i,j,a}^{Disp}\left( t \right)$ is one if a badger of age class *a* moves from social group *i* to social group *j* during time step *t*, and zero otherwise.

*Cattle farms*

Three health states were distinguished for cattle (*S*: susceptible, *E*: infected and non-infectious, detectable by screening tests without detectable post-mortem lesions, and *I*: infected and infectious, detectable by screening tests and presenting lesions detectable by post-mortem examination). The duration of the latency period (duration of state E) was estimated in Bekara et al. [46] at 3.6 months (Table 2).

We defined symmetric formulas to (1) and (2) for farms, with the exception of the 2^nd^ term of Equation 2 (movements of animals between populations), which was not transposed to farms, as cattle movements between farms simply implemented the data registered in the BDNI database. The health state of the moved animals was chosen randomly. Animals were kept on pastures (i.e. $1_{a}^{E}\left( t \right)=1$) from March to November, except for young heifers (0-1 years old) in dairy farms. Following the simplifying hypotheses made in Bekara et al. [46], we assumed that *M. bovis* transmission between cattle always occurred between animals of the same batch. However, because of the aging of animals, the composition of the batch changed every year. Animal transfers between batches then allowed *M. bovis* to spread between batches. In addition, the between-cattle transmission parameter, estimated in [46], was higher when animals were kept inside buildings. The model equations describing the within-farm part of the infectious process can be found in [46].

**Table 2. Model parameters for the cattle metapopulation.**

| **Notation** | **Description** | **Value** | **Source** |
| --- | --- | --- | --- |
| $\mu_{dairy}$ | Culling rate in dairy and mixed herds | 0.33 year^-1^ | BDNI |
| $\mu_{beef}$ | Culling rate in beef herds and in other herds | 0.31 year^-1^ | BDNI |
| ${Se}_{SITT}$ | SITT sensitivity | 0.91 | [74] |
| ${Sp}_{SITT}$ | SITT specificity | 0.91 | [74] |
| ${Se}_{SICTT}$ | SICTT sensitivity | 0.75 | [74] |
| ${Sp}_{SICTT}$ | SICTT specificity | 1 | [74] |
| ${Se}_{PCR}$ | PCR sensitivity (local lab.) | 0.86 | [74] |
| ${Sp}_{PCR}$ | PCR specificity (local lab.) | 1 | [74] |
| ${Se}_{NRL}$ | PCR sensitivity (NRL) | 1 | [38] |
| ${Sp}_{NRL}$ | PCR specificity (NRL) | 1 | [38] |
| ${Se}_{histo}$ | Histology sensitivity | 0.66 | [74] |
| ${Sp}_{histo}$ | Histology specificity | 1 | [74] |
| ${Se}_{cult}$ | Bacterial culture sensitivity | 0.74 | [74] |
| ${Sp}_{cult}$ | Bacterial culture specificity | 1 | [74] |
| ${Se}_{slaug}$ | Sensitivity of necropsy (meat inspection) | 0.71 | [74] |
| ${Sp}_{slaug}$ | Specificity of necropsy (meat inspection) | 1 | [74] |
| ${Se}_{streng}$ | Sensitivity of necropsy (detailed) | 0.96 | [74] |
| ${Sp}_{streng}$ | Specificity of necropsy (detailed) | 1 | [74] |
| ${Se}_{IF\gamma}$ | Sensitivity if gamma-interferon test | 0.55 | [74] |
| ${Sp}_{IF\gamma}$ | Specificity if gamma-interferon test | 0.99 | [74] |
| ${Se}_{sero}$ | Sensitivity of serological test | 0.60 | [74] |
| ${Sp}_{sero}$ | Specificity of serological test | 0.93 | [74] |
| $\alpha$ | Inverse of the duration of state E | 0.28 month^-1^  [0.13 – 0.56] | [46] |
| $\beta_{W}^{C}$ | Transmission parameter between cattle placed on the same pasture | 0.08 month^-1^  [0.01 – 0.32]^a^ | [46] |
| $\varepsilon_{N}^{C}$ | Proportion of within-group FOI exerted on animals of neighbouring pastures | estimated |  |
| $\beta_{E}^{c}$ | Transmission parameter from a contaminated pasture | estimated |  |

BDNI: “Base de Données Nationale d’Identification”, the database of the French cattle tracing system. SITT: Single Intradermal Tuberculin Test; SCTT: Single Intradermal Comparative Cervical Tuberculin Test; PCR: Polymerase Chain Reaction; NRL: National Reference Laboratory. ^a^Estimate obtained by Bekara et al. [46] when cattle are on pasture; the within-farm transmission model uses a higher value when animals are inside buildings (0.43 [0.16-0.84]).

**BTB surveillance and control**

*Badger groups*

In badgers, bTB surveillance and control was based on trapping, which started in 2012. The trapping effort required from field agents varied according to the epidemiological local situations. Three situations were distinguished by animal health authorities:

1. municipalities with at least one cattle detected outbreak, where the trapping effort was the highest;
2. municipalities with no detected cattle outbreak, but with cattle outbreaks in the neighbouring municipalities: the trapping effort was moderate;
3. Other municipalities, where the trapping effort was lower.

The parameterization of trapping probabilities is described in Additional file 2. The distribution of municipalities in categories (A), (B) and (C) could change at each time step, depending on the evolution of the situation in farms. We assumed that trapping operations were performed from March to September, and that trapping could concern all the animals, whatever their age, although cubs be trapped only from May, when they leave the sett. The number of trapped animals by health state and age was thus randomly chosen at each time step, for each badger group. We assumed that all the trapped animals were necropsied and tested for the presence of *M. bovis*. The specificity of the test was assumed perfect. Because of the often poor state of preservation of the samples, the sensitivity was lower (Table 1).

*Cattle farms*

The screening of cattle farms was performed every two years in the Landes department and every three years in the Pyrénées-Atlantiques (PA) department, except for farms that had been infected in the preceding years (8 in Landes and 16 in PA), where the screening was annual. From 2012, the screening became annual in municipalities with detected outbreaks. The surveillance protocol differed according to the type of farm:

- In dairy farms, the screening was performed using SICTT (Single Intradermal Comparative cervical Tuberculin Test) and positive animals were slaughtered for a detailed necropsy examination. In case of presence of *M. bovis* (based on a combination of histology, culture, PCR and on a confirmation by the National Reference Laboratory [NRL]), the entire herd was depopulated. Otherwise the herd was screened again 2 months later using SICTT. In case of presence of *M. bovis* the herds was depopulated; otherwise it recovered its bTB-free status.
- In other farms, SITT (Single Intradermal Tuberculin Test) was used for screening. If positive results were confirmed using SICTT 2 months later, and positive animals were slaughtered for a detailed necropsy examination. In case of presence of *M. bovis*, the entire herd was depopulated. Otherwise it recovered its bTB-free status.

From 2012, the screening used SICTT in municipalities with detected outbreaks. Routine abattoir surveillance was performed on all the slaughtered animals, and detected lesions were tested for the presence of *M. bovis* using a combination of histology, PCR and culture. In case of positive results, both of the above surveillance protocols were implemented in the animal’s farm of origin. From 2016, the entire depopulation of infected herd could be replaced by a selective slaughter protocol: farms recovered their bTB-free status after three negative whole-herd screenings, performed three months apart. The first two screenings used SICTT in dairy farms (and in farms from municipalities with detected outbreaks) and SITT in other farms. The third screening was always performed using SICTT. In case of positive result, the animal was tested using a combination of gamma interferon test and serology. Positive animals were slaughtered for a detailed necropsic examination, in case of the presence of *M. bovis* (based on a combination of histology, culture and PCR). The sensitivities and specificities of diagnostic test, were obtained from literature (Table 2). We assumed they were not influenced by the genotype of *M. bovis*.

**References**

All bibliographic reference numbers used above refer to the complete references list provided in the main manuscript.
