# Supplementary material for: Analysis of a multi-type resurgence of Mycobacterium bovis in cattle and badgers in Southwest France, 2007-2019
Source: Vet Res. 2023 May 3;54:41. doi: 10.1186/s13567-023-01168-8 (PMC10158257; doi:10.1186/s13567-023-01168-8)
Supplement: Supplementary file 2 — Additional file 2: Calibration of the parameters of badger population dynamics. [file 13567_2023_1168_MOESM2_ESM.docx]

**Additional file 2. Calibration of the parameters of badger population dynamics**

Three parameters were calibrated: *K* (the threshold number of adult and subadult badgers used for the calculation of the monthly dispersal and mortality probabilities), $\rho$ (the yearly probability of reproduction in a social group), and *δ* (the individual monthly dispersion rate) (see Additional file 1 for details).

We used the results of a recent field study conducted in France [43], in 13 contrasted sites of 50 km^2^. In this study, the authors estimated the average values of six variables describing the French badger population: the proportion of occupied sett clusters without reproduction (0.51, based on the observation of cubs) or with reproduction (0.13), and the average number of animals in sett clusters with reproduction (4.46 animals of which 1.95 adults or subadults) or without reproduction (1.7 animals of which 1.35 adults or subadults).

We used adaptive population Monte-Carlo approximate Bayesian computation (APMC-ABC) [50] to calibrate three parameters: the threshold number of adult and subadult badgers in the sett cluster, below which mortality probability decreases, and above which dispersion probability increases in the social group (*K*), the yearly probability of reproduction in a social group of at least two adults ($\rho$) and the dispersion probability (*δ*). The prior distribution of *K* was *U*(0.2, 2) (*U*: uniform distribution), considering, for the upper limit, that the effect of group size on dispersal and mortality rates should be noticeable above two animals (allowing for reproduction); and using one-tenth of the upper limit for the lower limit. For $\rho$, an arbitrary large prior was used: *U*(0.2, 0.8). For *δ*, the prior distribution was *U*(0.001, 0.02), using as upper limit the mean of the values reported by Woodroffe et al. [75] in Bristol and Speyside areas, UK where badger density (respectively 5.5 and 2.2 adults per km²) were slightly higher than in our study area, and an arbitrary low value for the lower bound.

**Figure 1. Posterior distribution of the threshold number of adult and sub-adult badgers in the sett cluster, below which mortality probability decreases, and above which dispersion probability increases (*K*), the dispersion rate (*δ*), and the yearly probability of reproduction in a social group of at least two adults (**$\boldsymbol{\rho}$**) (stars: bounds of the prior distribution)**


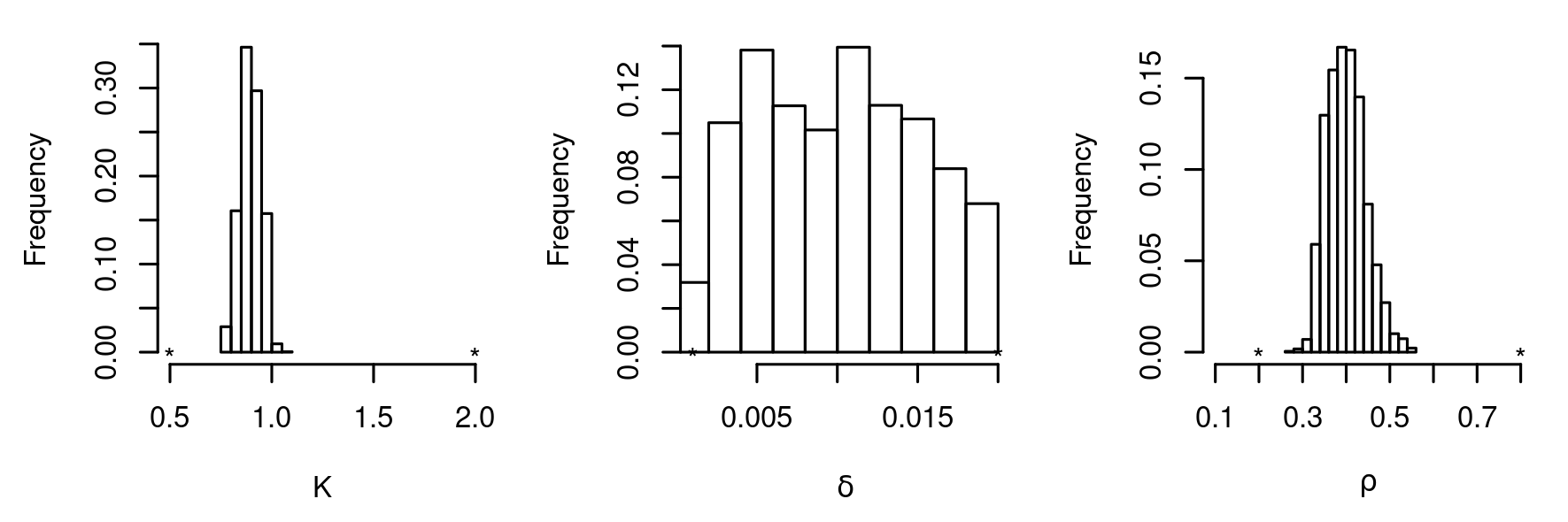


The model was used to simulate badger population dynamics (without bTB) conditional to the values of *K*, $\rho$ and *δ* drawn from prior distributions. Simulations started by randomly choosing, among the 1750 sett clusters, 13% of occupied clusters without reproduction and 51% with reproduction, other clusters being left empty. There was initially a single adult in sett clusters without reproduction, whereas for those with reproduction there were initially two adults, one subadult and a number of cubs drawn from the distribution of litter sizes (Additional file 1: Table 1). Simulations lasted 60 years and allowed computing the six summary statistics described above, averaged over the last 10 simulated years.

As the badger density is considered globally stable in France (although it slightly increased in the southern part of the study area between 2004 and 2012) [76], a seventh summary statistic was used: the largest value of the time trend of the 6 other statistics, computed for the last 10 simulated years, the target value being 0. The recommended settings [50] were used for the ABC-APMC algorithm, and we used 1000 particles to build the posterior distributions.

Posterior distributions (Figure 1) showed that, contrary to *K* and $\rho$, the data used for calibration did not allow estimating precisely the dispersion probability (*δ*), for which the prior and posterior distributions were close. However, we used this posterior distribution in subsequent calculations, as it allowed to take into account the uncertainty about the value of this parameter.

**Figure 2. Predicted evolution during the last ten simulated years (orange lines, 100 simulations) and values (plain blue line: estimate, dashed blue lines: confidence interval) reported in [43] of the proportion of occupied sett clusters (CS) without or with reproduction, and the average number of adults and animals in both categories of sett clusters.**


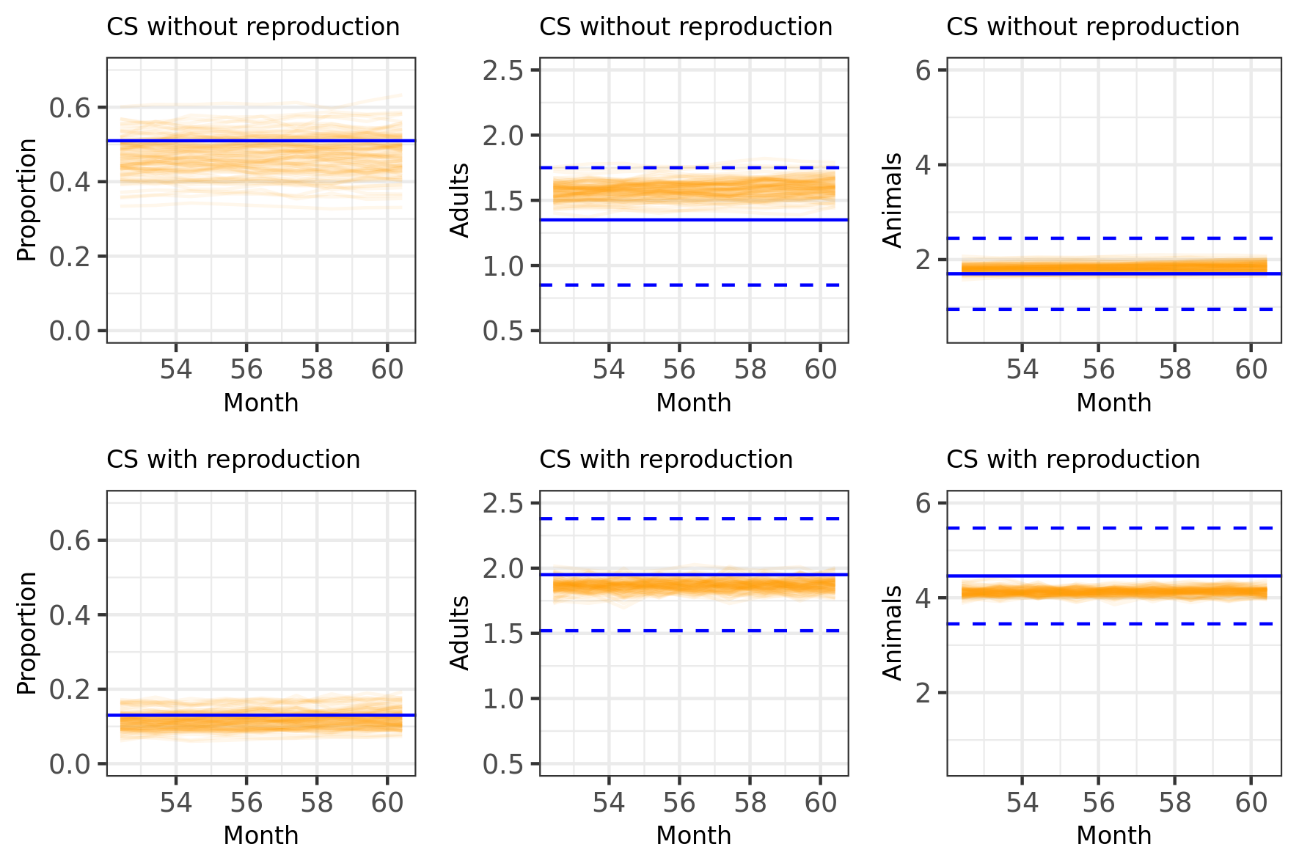


Model fit was satisfactory as in 100 simulations, population dynamics appeared stable, and reproduced the observed data (Figure 2). The final state produced by these 100 simulations was used as 100 possible initial states of the entire badger metapopulation. In these initial states, the average proportion of occupied clusters of setts was 0.58 (range: 0.40-0.76), corresponding to 1011 badger groups (range: 701-1329) and to a density of 0.37 badger groups per km^2^ (range: 0.26-0.49 groups per km^2^). Trapping rates were computed for each of the 100 initial states using as denominator the simulated number of adults and subadults in each commune, and as numerator the actual number of badgers trapped and culled in each commune. Because the trapping effort of field agents was different, we distinguished three situations (and estimated three distinct trapping rates for each of the 100 initial states): communes with ≥1 farms reported infected, neighboring communes, and other communes (in each case, only the first year with trapping was considered).

**References**

All bibliographic reference numbers used above refer to the complete references list provided in the main manuscript.
