# Supplementary material for: Analysis of a multi-type resurgence of Mycobacterium bovis in cattle and badgers in Southwest France, 2007-2019
Source: Vet Res. 2023 May 3;54:41. doi: 10.1186/s13567-023-01168-8 (PMC10158257; doi:10.1186/s13567-023-01168-8)
Supplement: Supplementary file 3 — Additional file 3: Density of cattle and reconstructed badger density, at the beginning of the study period, in subareas wider than 30 km2. [file 13567_2023_1168_MOESM3_ESM.docx]

**Additional file 3. Density of cattle and reconstructed badger density, at the beginning of the study period, in subareas wider than 30 km^2^**

| **Subarea**^1^ | **Badger density**^2^ | **Cattle density**^3^ | **Surface (km^2^)** |
| --- | --- | --- | --- |
| 02 | 0.96 (0.58-1.36) | 25.4 | 424 |
| 26 | 0.89 (0.56-1.37) | 38.6 | 500 |
| 32 | 0.83 (0.26-1.24) | 54.1 | 108 |
| 08 | 0.81 (0.30-1.50) | 59.1 | 38.1 |
| 16 | 0.76 (0.39-1.19) | 24.6 | 339 |
| 04 | 0.75 (0.26-1.26) | 30.7 | 80 |
| 18 | 0.68 (0.32-1.13) | 35.6 | 204 |
| 28 | 0.65 (0.21-1.20) | 59.0 | 103 |
| 01 | 0.62 (0.36-1.06) | 22.2 | 196 |
| 03 | 0.60 (0.16-1.01) | 20.5 | 68 |
| 13 | 0.59 (0.16-1.08) | 56.7 | 63 |
| 10 | 0.57 (0.18-1.09) | 18.2 | 128 |
| 11 | 0.53 (0.19-0.88) | 22.3 | 255 |

^1^Subareas are ranked by decreasing badger density. ^2^Median number of animals per km^2^ in 100 initial states of the metapopulation, reconstructed by simulation. Brackets: 2.5% and 97.5% percentiles. ^3^Number of cows per km^2^, according to BDNI database.
