# Supplementary material for: Analysis of a multi-type resurgence of Mycobacterium bovis in cattle and badgers in Southwest France, 2007-2019
Source: Vet Res. 2023 May 3;54:41. doi: 10.1186/s13567-023-01168-8 (PMC10158257; doi:10.1186/s13567-023-01168-8)
Supplement: Supplementary file 4 — Additional file 4: Estimation of M. bovis transmission parameters. [file 13567_2023_1168_MOESM4_ESM.docx]

**Additional file 4. Estimation of *M. bovis* transmission parameters**

Five parameters were driving the different *M. bovis* transmission pathways: between badgers of the same social group ($\beta_{W}^{B}$), between badgers of neighboring social groups ($\varepsilon_{N}^{B}$), from a pasture contaminated by infected cattle to the badgers visiting the pasture ($\beta_{E}^{B}$), between cattle of neighboring farms ($\varepsilon_{N}^{C}$), and from a pasture contaminated by infected badgers to the cattle using the pasture ($\beta_{E}^{C}$). We assumed these transmission parameters were not influenced by the genotype of *M. bovis*. The prior distributions were defined as follows:

- Transmission parameter between badgers of the same social group ($\beta_{W}^{B}$): the prior distribution was *U*(4.3 10^-3^, 0.25). We used the probability, in a group of two badgers, one free and one infected, that the free individual would be infected after 24 months. Considering a range of 0.05-0.95 for this probability *q*, we obtained the bounds of the prior distribution by solving $q=1-e^{-24 {\beta_{W}^{B}}/2}$.
- Ratio of the transmission parameter between badgers of neighboring social group to the within-group transmission parameter ($\varepsilon_{N}^{B}$): the prior distribution was *U*(10^-6^, 0.5). The lower bound was set to an arbitrary low value. We set the upper bound by assuming that the intensity of contact between badgers of neighboring social should be less than half the intensity of contact between badgers of the same social group.
- Transmission parameter from a contaminated pasture to badger visiting that pasture ($\beta_{E}^{B}$): the prior distribution was *U*(10^-6^, 10^-1^). The lower bound was set to an arbitrary low value. We set the upper bound by assuming that the probability of a badger becoming infected after 24 months of exposure to cattle-contaminated pasture should not exceed the probability of becoming infected if placed in contact with an infectious badger during the same period. Solving the equation $1-e^{-24 \beta_{E}^{B}}\leq1-e^{-24 {\beta_{W}^{B}}/2}$, we set the upper bound of $\beta_{E}^{B}$ to half that of $\beta_{W}^{B}$, rounded to 0.1.
- Ratio of the transmission parameter between cattle of neighboring herds to the within-herd transmission parameter ($\varepsilon_{N}^{C}$): the prior distribution was *U*(10^-4^, 10^-1^). We set the lower bound to an arbitrary low value. However, assuming the intensity of contacts between cattle placed on neighboring pastures was higher than between badgers living in neighboring social groups, we set this lower bound to a slightly higher value than for $\varepsilon_{N}^{B}$. The upper bound was the transmission parameter between cattle sharing the same pasture, estimated 0.08 month-1 in Bekara et al. [46], and rounded to 0.1.
- Transmission parameter from a contaminated pasture to cattle placed on that pasture ($\beta_{E}^{C}$): the prior distribution was *U*(10^-6^, 10^-3^). The lower bound was set to an arbitrary low value. Concerning the upper bound, we calculated the probability of a cattle becoming infected in a herd where a single animal is infectious: $1-e^{-12 {\beta_{W}^{C}}/N}$, where N is the size of the herd. We assumed this probability to be greater than the probability that a cattle would become infected if placed on a badger-contaminated pasture for the same length of time: $1-e^{-12 \beta_{E}^{C}}$. Using for $\beta_{W}^{C}$ the value estimated by Bekara et al., (0.08 month-1) [46], and for N the average size of dairy (76.2 animals) or beef herds (54.6 animals) resulted in an upper bound of 0.001 for the prior distribution of $\beta_{E}^{C}$.

We defined 22 summary statistics, which described the time evolution of bTB incidence (according to three periods: 2007-2011, 2012-2015, 2016-2019), the geographic distribution of the infection (in the four most affected subareas), the diversity of genotypes (focusing on the two types observed in both species), the within-group incidence in badgers, and the occurrence of breakdowns in farms (Table 1).

**Table 1. Summary statistics used for the parameter estimation.**

| **Summary statistic** | **Observed value** | **Predicted value**  **[95% CI]** |
| --- | --- | --- |
| Number of farms detected infected in 2007-2011 | 24 | 28.74 [11.00-65.00] |
| Number of farms detected infected in 2012-2015 | 45 | 79.86 [3.00-275.00] |
| Number of farms detected infected in 2016-2019 | 82 | 63.28 [1.00-237.00] |
| Number of badger social groups detected infected in 2012-2015 | 39 | 30.64 [2.00-94.00] |
| Number of badger social groups detected infected in 2016-2019 | 26 | 26.54 [1.00-79.00] |
| Number of farms detected infected in subarea 2^a^ | 40 | 32.73 [1.00-116.00] |
| Number of farms detected infected in subarea 26^a^ | 27 | 50.95 [1.00-219.00] |
| Number of farms detected infected in subarea 11^a^ | 20 | 28.31 [3.00-88.00] |
| Number of farms detected infected in subarea 16^a^ | 14 | 19.46 [1.00-81.00] |
| Simpson’s diversity index of genotypes detected in cattle | 0.55 | 0.57 [0.15-0.83] |
| Number of farms detected infected by G1 | 90 | 75.56 [2.00-283.00] |
| Number of farms detected infected by G2 | 45 | 41.35 [0.00-230.00] |
| Simpson’s diversity index of genotypes detected in badgers | 0.27 | 0.50 [0.00-0.78] |
| Number of badger social groups detected infected by G1 | 47 | 20.40 [0.00-76.00] |
| Number of badger social groups detected infected by G2 | 10 | 13.52 [0.00-71.00] |
| Number of badger social groups detected infected in subarea 2^a^ | 23 | 14.08 [0.00-53.00] |
| Number of badger social groups detected infected in subarea 26^a^ | 13 | 19.87 [0.00-76.00] |
| Number of badger social groups detected infected in subarea 11^a^ | 10 | 5.72 [0.00-22.02] |
| Number of badger social groups detected infected in subarea 16^a^ | 11 | 7.06 [0.00-31.00] |
| Mean number of badgers detected infected per social group | 1.14 | 1.28 [1.00-1.60] |
| Number of farms with one bTB episode during the study period | 132 | 80.08 [16.00-201.00] |
| Number of farms with two bTB episodes during the study period | 8 | 24.28 [1.00-81.00] |

^a^See Figure 1A in the main text

We used the recommended settings [50] for the ABC-APMC algorithm, and a total of 5,000 particles to build the posterior distributions shown in Figure 1.

**Figure 1. Posterior distributions of parameters driving *M. bovis* transmission: from a contaminated pasture (badgers:** $\boldsymbol{\beta}_{\boldsymbol{E}}^{\boldsymbol{B}}$**, cattle:** $\boldsymbol{\beta}_{\boldsymbol{E}}^{\boldsymbol{C}}$**), between neighboring populations (badgers:** $\boldsymbol{\varepsilon}_{\boldsymbol{N}}^{\boldsymbol{B}}$**, cattle:** $\boldsymbol{\varepsilon}_{\boldsymbol{N}}^{\boldsymbol{C}}$**), and within a badger social group (**$\boldsymbol{\beta}_{\boldsymbol{W}}^{\boldsymbol{B}}$**) (dashed lines: bounds of the prior distributions).**


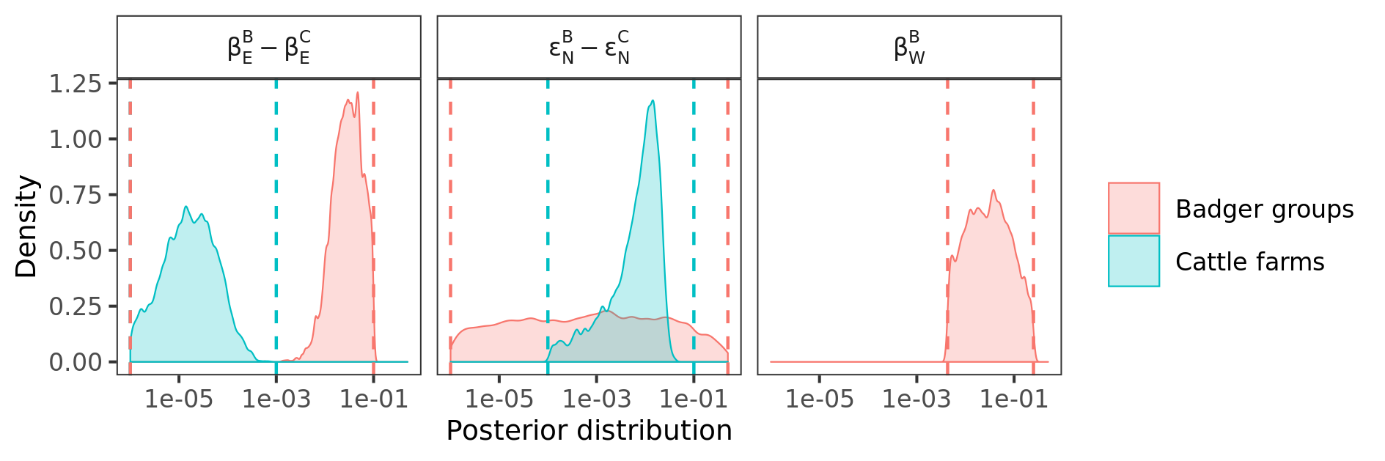


**References**

All bibliographic reference numbers used above refer to the complete references list provided in the main manuscript.
