# Supplementary material for: Analysis of a multi-type resurgence of Mycobacterium bovis in cattle and badgers in Southwest France, 2007-2019
Source: Vet Res. 2023 May 3;54:41. doi: 10.1186/s13567-023-01168-8 (PMC10158257; doi:10.1186/s13567-023-01168-8)
Supplement: Supplementary file 5 — Additional file 5: Choice of the bTB initialization scenario. [file 13567_2023_1168_MOESM5_ESM.docx]

**Additional file 5. Choice of the bTB initialization scenario**

Nine scenarios concerning the initial presence of *M. bovis* were considered, assuming that the two molecular types reported in cattle and badgers (G1 and G2) were already present at the beginning of the study period in one or both species. We compared these scenarios using a model choice procedure proposed by Pudlo et al. [51], and specifically designed for inference using ABC. We ran 10 000 simulations for each scenario, using the prior distributions (Additional file 3), to set the values of $\beta_{W}^{B}$, $\varepsilon_{N}^{B}$, $\beta_{E}^{B}$, $\varepsilon_{N}^{C}$, and $\beta_{E}^{C}$, and computed for each simulation the 22 summary statistics (Additional file 3). We trained a random forest (1000 trees) using this dataset to predict, from the simulated summary statistics, the scenario used. We then used this random forest to predict the scenario corresponding to the observed values of the summary statistics. The result was the distribution of classification votes among the nine scenarios, and we selected the scenario with the highest number of votes for parameter estimation and model exploitation (Table 1). As the posterior probability (computed using the trained random forest) that the true scenario is the selected scenario was low (0.25), we investigated in the sensitivity analysis the eight remaining scenarios.

**Table 1. Choice of the bTB initialization scenario for G1 and G2**

| **Initial infection** | | **Votes** |
| --- | --- | --- |
| **G1** | **G2** | **(1000 classification trees)** |
| Cattle | Both | 0.228 |
| Cattle | Badgers | 0.124 |
| Badgers | Both | 0.122 |
| Both | Both | 0.113 |
| Cattle | Cattle | 0.104 |
| Both | Badgers | 0.094 |
| Badgers | Badgers | 0.094 |
| Both | Cattle | 0.063 |
| Badgers | Cattle | 0.058 |

**References**

The bibliographic reference number used above refers to the complete references list provided in the main manuscript.
