# Supplementary material for: Analysis of a multi-type resurgence of Mycobacterium bovis in cattle and badgers in Southwest France, 2007-2019
Source: Vet Res. 2023 May 3;54:41. doi: 10.1186/s13567-023-01168-8 (PMC10158257; doi:10.1186/s13567-023-01168-8)

**Additional file 6. Model fit to observed data along the three dimensions of the model: genotype, space (subarea) and time period**

**Figure 1. Predicted (colored dots, bars: 95% CI) and observed (black dots) variations of the apparent incidence in cattle and badgers according to genotype and subarea.**


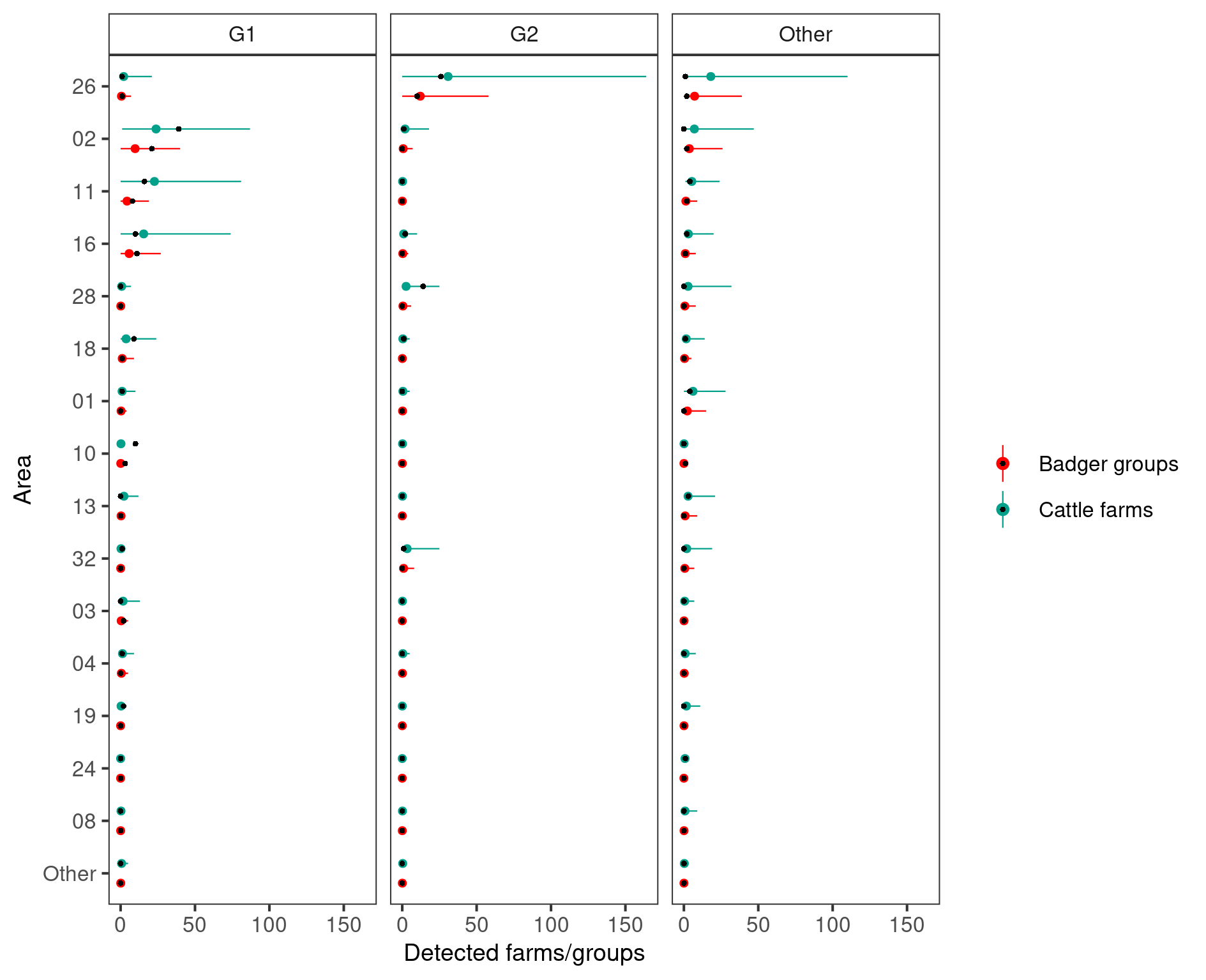


**Figure 2. Predicted (colored dots, bars: 95% CI) and observed (black dots) variations of the apparent incidence in cattle and badgers according to period and subarea.**


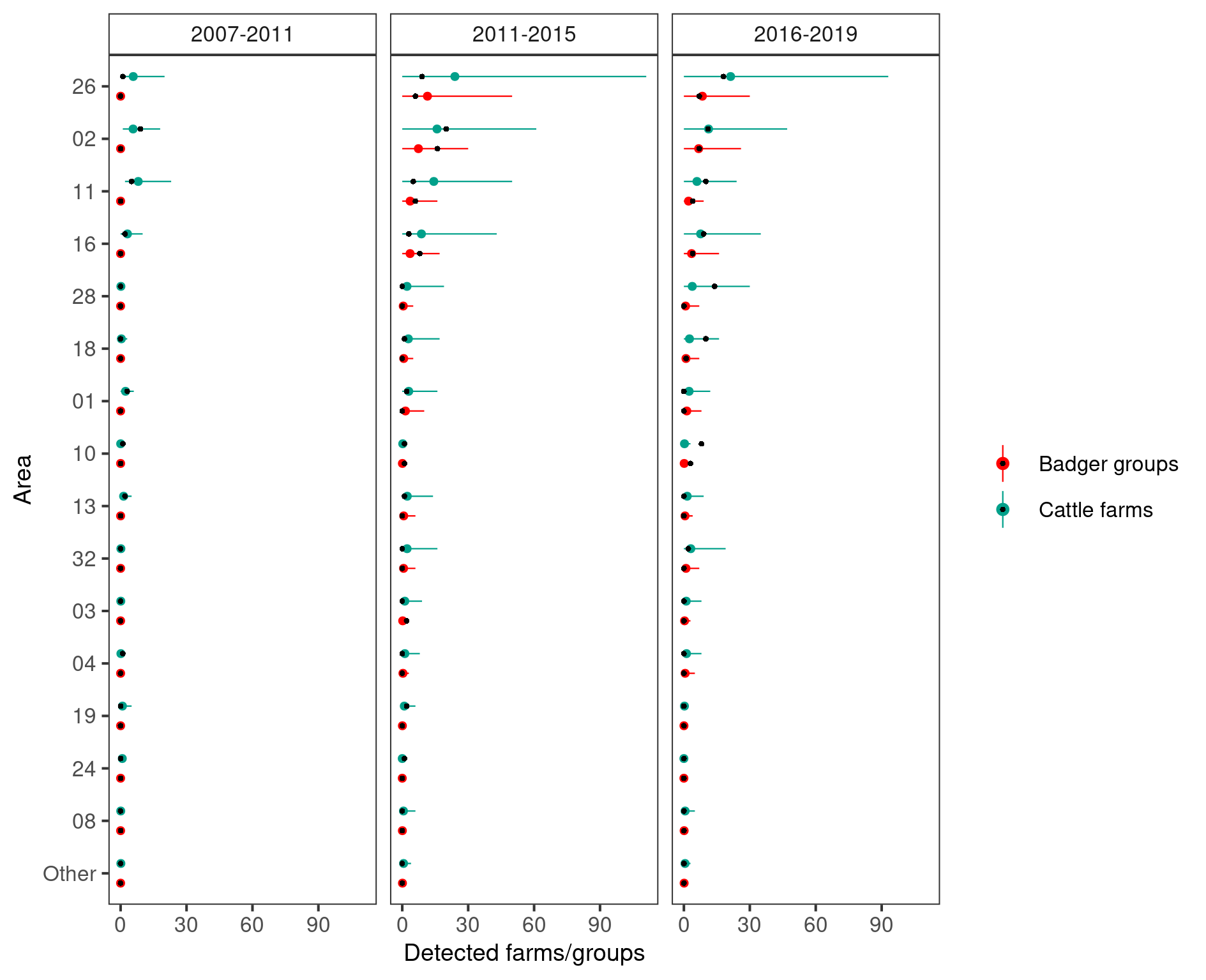


**Figure 3. Predicted (plain lines, colored areas: 95% CI) and observed (dashed lines) variations of the apparent incidence in cattle and badger according to genotype and period.**


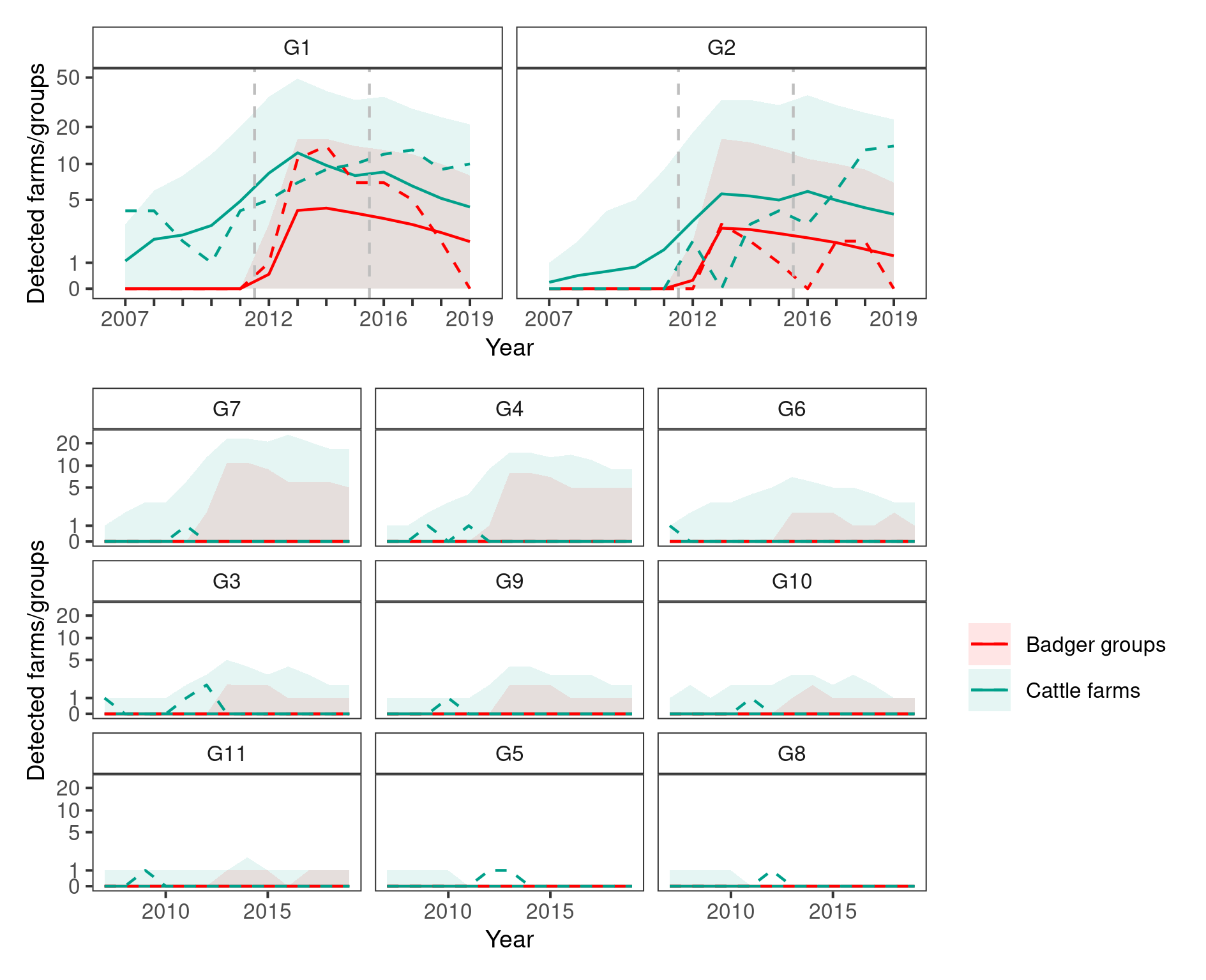

Supplement: Supplementary file 6 — Additional file 6: Model fit to observed data along the three dimensions of the model: genotype, spaceand time period. [file 13567_2023_1168_MOESM6_ESM.docx]
