# Supplementary figures and images for: Analysis of a multi-type resurgence of Mycobacterium bovis in cattle and badgers in Southwest France, 2007-2019
Source: Vet Res. 2023 May 3;54:41. doi: 10.1186/s13567-023-01168-8 (PMC10158257; doi:10.1186/s13567-023-01168-8)

**Additional file 7. Geographic variations of the reconstructed incidence (A) and prevalence (B)**


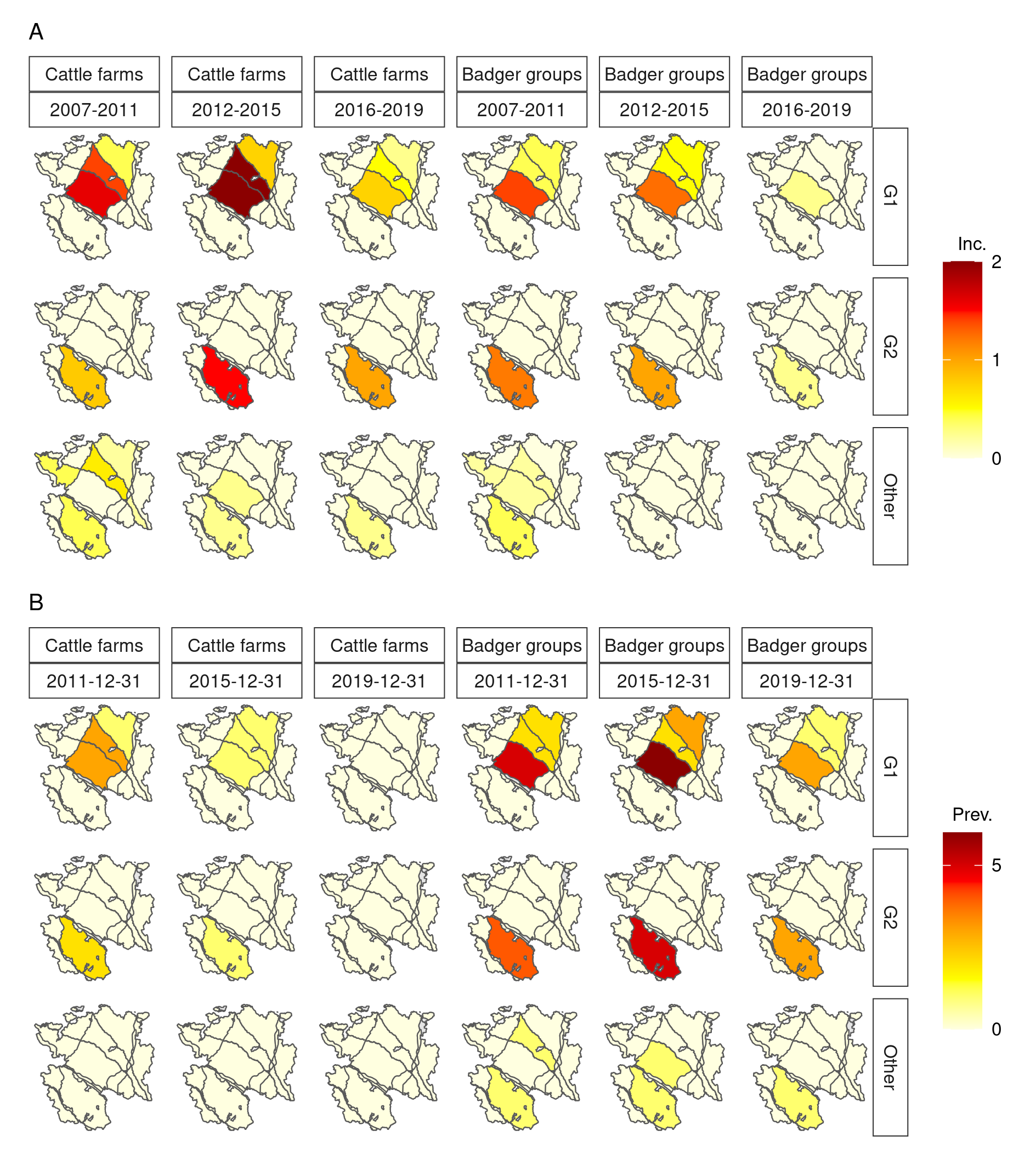

Supplement: Supplementary file 7 — Additional file 7: Geographic variations of the reconstructed incidence and prevalence. [file 13567_2023_1168_MOESM7_ESM.docx]
