# Supplementary material for: Analysis of a multi-type resurgence of Mycobacterium bovis in cattle and badgers in Southwest France, 2007-2019
Source: Vet Res. 2023 May 3;54:41. doi: 10.1186/s13567-023-01168-8 (PMC10158257; doi:10.1186/s13567-023-01168-8)
Supplement: Supplementary file 8 — Additional file 8: Sensitivity analysis. [file 13567_2023_1168_MOESM8_ESM.docx]

**Additional file 8. Sensitivity analysis**

**Figure 1. Variations of the posterior distributions (dots: mean value, lines: 95% credibility interval) of parameters driving *M. bovis* transmission according to the initial conditions, the disease-induced mortality rate (**$\boldsymbol{\mu}_{\boldsymbol{d}}$**), the sensitivity of diagnostic tests used in badgers (*Se*), and the duration of *M. bovis* survival on pastures (*s*).**


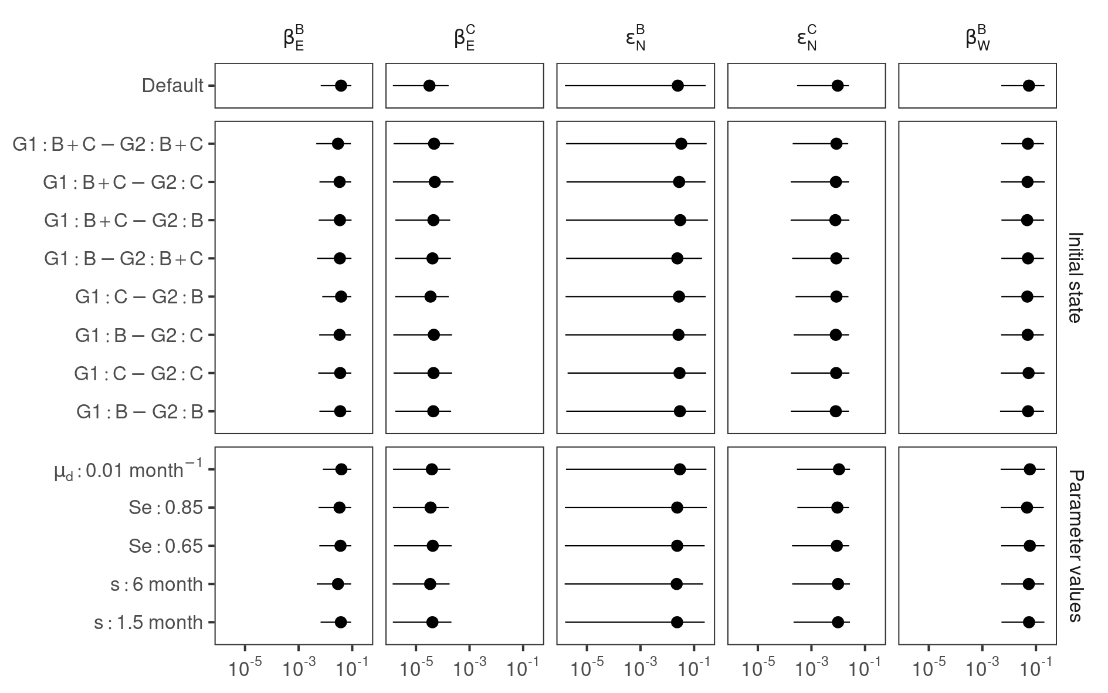


Default: default parameterization, i.e. G1 initially present in cattle only, G2 in cattle and badgers, no disease induced mortality parameter ($\mu_{d}=0)$, Sensitivity of diagnostic tests used in wildlife (Se) of 0.75, Duration of *M. bovis* survival on pastures of three months. Transmission parameters: from a contaminated pasture (badgers: $\beta_{E}^{B}$, cattle: $\beta_{E}^{C}$), between neighboring populations (badgers: $\varepsilon_{N}^{B}$, cattle: $\varepsilon_{N}^{C}$), and within a badger social group ($\beta_{W}^{B}$). Initial conditions: presence of the two molecular types detected in cattle and badgers (G1 and G2) in badgers only (B), in cattle only (C), or in both species (B+C).

**Figure 2. Variations of the overall and partial reproduction numbers according to the initial conditions, the disease-induced mortality rate (**$\boldsymbol{\mu}_{\boldsymbol{d}}$**), the sensitivity of diagnostic tests used in badgers (*Se*), and the duration of *M. bovis* survival on pastures (*s*).**

**
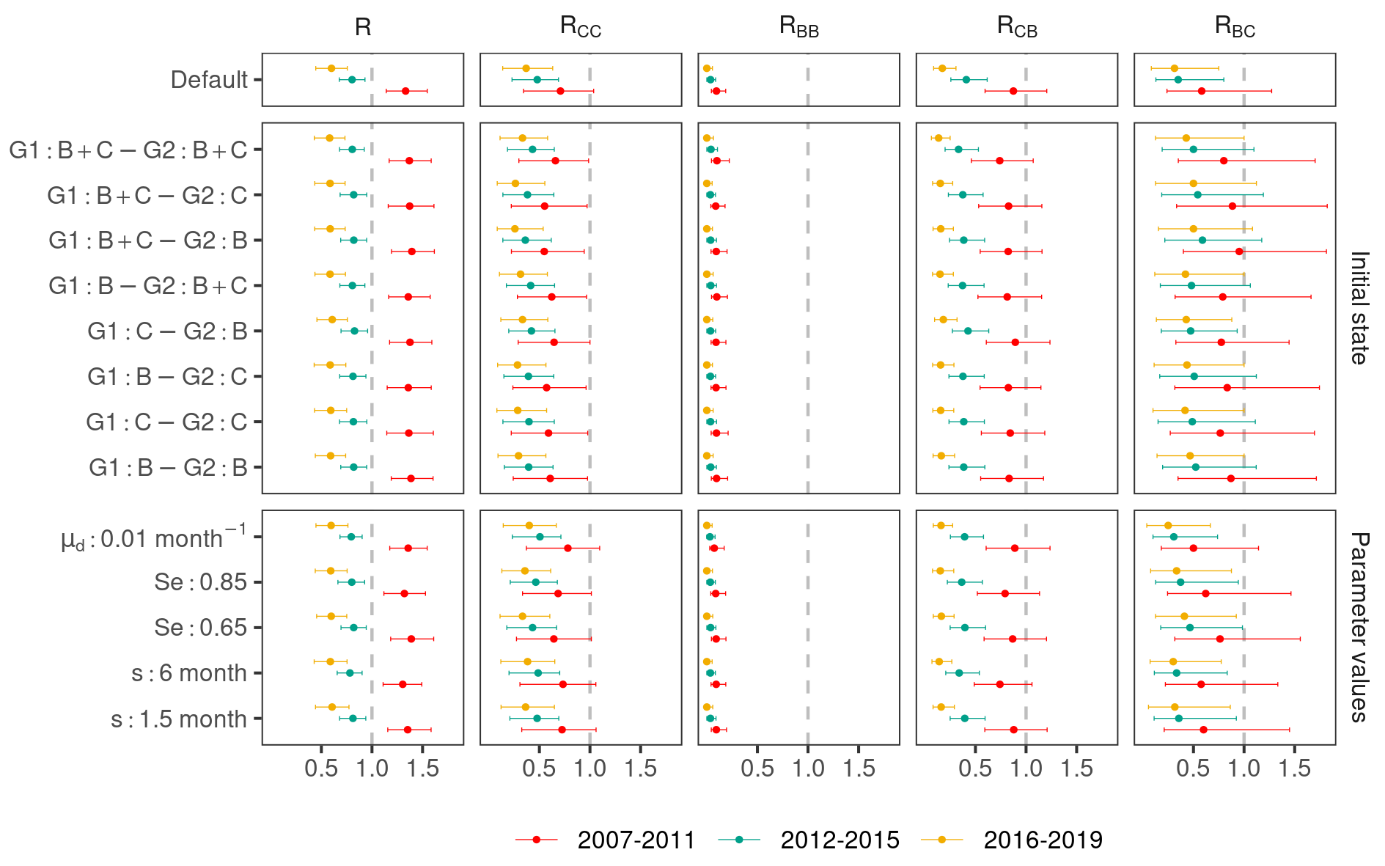
**

*R*: overall reproduction number. *R_CC_*: partial reproduction number between cattle farms, *R_CB_*: from cattle farms to badger groups, *R_BB_*: between badger groups, *R_BC_*: from badger groups to farms. Default: default parameterization, i.e. G1 initially present in cattle only, G2 in cattle and badgers, no disease induced mortality parameter ($\mu_{d}=0)$, Sensitivity of diagnostic tests used in wildlife (Se) of 0.75, Duration of *M. bovis* survival on pastures of three months. Initial conditions: presence of the two genotypes detected in cattle and badgers (G1 and G2) in badgers only (B), in cattle only (C), or in both species (B+C). Dots: median values; error bars: the inter-quartile range.
